# Mpox coinfections and clinical manifestation in Africa: a systematic review and meta-analysis

**DOI:** 10.3389/fsysb.2026.1795422

**Published:** 2026-05-07

**Authors:** Fabrice Zobel Lekeumo Cheuyem, Chabeja Achangwa, Lionel Berthold Keubou Boukeng, Rick Tchamani, Jessica Davies, Christian Noël Malaka, Armel Evouna Mbarga

**Affiliations:** 1 Department of Public Health, Faculty of Medicine and Biomedical Sciences, The University of Yaoundé 1, Yaoundé, Cameroon; 2 Department of Public Health, Faculty of Medical Sciences, University of West Indies, Bridgetown, Barbados; 3 Direction of Disease, Epidemics and Pandemics Control, Ministry of Public Health, Yaoundé, Cameroon; 4 Department of Public Health and Social Sciences, Faculty of Medicine and Pharmaceutical Sciences of Sangmelima, University of Ebolowa, Ebolowa, Cameroon; 5 Central Africa University Institute of Excellence, Yaoundé, Cameroon; 6 International Association of Student Surgical Societies, Eastern Cape, South Africa; 7 Researcher and Head of the Genomic Surveillance Platform, Institut Pasteur, Bangui, Central African Republic

**Keywords:** Africa, coinfection, HIV, meta-analysis, molecular epidemiology, mpox, systematic review, varicella-zoster virus

## Abstract

**Background:**

Mpox remains a significant public health challenge in Africa, where endemic transmission persists alongside a high burden of other infectious diseases. Although the epidemiology and clinical impact of coinfections with human immunodeficiency virus (HIV) and varicella-zoster virus (VZV) remain poorly understood across the continent, these coinfections may influence clinical presentation, disease severity, and diagnostic accuracy. This aimed to describe the coinfections (VZV and HIV) patterns among confirmed mpox cases and characterize the trend of clinical presentation of mpox patients in Africa.

**Methods:**

Following PRISMA guidelines, we registered our protocol in PROSPERO (CRD420251133960) and conducted a systematic review and meta-analysis. We searched multiple electronic databases and grey literature through 27 February 2025, identifying observational studies from Africa that reported mpox coinfections (VZV and/or HIV) and associated clinical symptoms. Random-effects models were used to calculate pooled prevalence, while subgroup analyses and meta-regression explored sources of heterogeneity across WHO regions, countries, study designs, settings, and participant types.

**Results:**

A total of 23 studies conducted across African countries were included. The pooled prevalence of VZV–mpox coinfection was 8.73% (95% CI: 2.05–30.43; 10 studies; n = 2,681; *I* ^2^ = 75.7%), while HIV–mpox coinfection prevalence was 4.29% (95% CI: 1.78–9.96; 9 studies; n = 1,939; *I* ^2^ = 88.4%), both of which had significant heterogeneity. Coinfections were far more prevalent in hospital-based environments than in community-based research. The rash was observed across all clades, but the clinical manifestations varied by viral clades, with clades I and Ia linked to more severe systemic symptoms than clade II.

**Discussions:**

HIV and VZV coinfections with mpox pose a major yet possibly underestimated burden in Africa and are linked to more severe clinical presentations, particularly in hospital environments. The necessity of including clinical, epidemiological, and genomic in mpox monitoring systems is underscored by observed clinical differences across clades. Improving patient management and outbreak preparedness across the continent requires strengthening diagnostic capacity and routinely screening for coinfections.

**Systematic Review Registration:**

https://www.crd.york.ac.uk/PROSPERO/view/CRD420251133960, identifier CRD420251133960.

## 1 Introduction

Mpox is a re-emerging zoonotic viral infection, caused by a stable DNA *Orthopoxvirus* belonging to the *Poxviridae* family, that has now become a significant global health concern (Sah et al., 2022; Africa CDC, 2024). During the 2022–2023 multicountry outbreak, more than 87,000 confirmed cases were reported globally, with the United States and Brazil ranking among the most affected countries, underscoring its potential for rapid international spread (Di Gennaro et al., 2022; WHO, 2022). In Africa, the epidemiological burden remains disproportionately high (Cheuyem et al., 2025b). In Central Africa, the Democratic Republic of the Congo (DRC) reported over 14,600 suspected cases and >500 deaths between 2023 and 2024, and Cameroon continues to register recurrent clusters across multiple regions (Bangwen et al., 2025; Brosius et al., 2025; Ebede et al., 2025). In West Africa, Nigeria has documented more than 1,400 suspected and confirmed cases since 2017, with resurgences in 2022 and 2023, while Ghana recorded confirmed outbreaks in 2022, including pediatric cases (Cadmus et al., 2024; WHO, 2024c; Cibenda et al., 2025). Coinfections with endemic pathogens particularly HIV, bacterial skin infections, sexually transmitted infections, and varicella-zoster virus (VZV) are widespread in these settings and may significantly modulate disease severity, transmission dynamics, and clinical outcomes (Hughes et al., 2021; Cheuyem et al., 2025a).

Mpox virus is classified into two main clades. Clade I, historically associated with Central Africa, is linked to higher virulence and has recently been subdivided into Ia and Ib, with Clade Ib emerging in the DRC in 2023 and demonstrating greater transmissibility (Iroezindu et al., 2023; Brosius et al., 2025). Clade II, predominant in West Africa, includes Clade IIb, which drove the global outbreak between 2022 and 2023 and is associated with milder clinical outcomes (WHO, 2022; Iroezindu et al., 2023). Classically, mpox presents with a febrile prodrome followed by a vesiculopustular rash and lymphadenopathy. However, clinical manifestations in Africa are often modified by co-existing infections (Hughes et al., 2021; Cheuyem et al., 2025a). These coinfections may significantly modulate disease severity, transmission dynamics, and outcomes. In resource-constrained settings, the overlapping burden of mpox and these infectious diseases presents unique challenges for clinical management and public health control, making research into coinfection dynamics both urgent and essential (Hughes et al., 2021; Cheuyem et al., 2025a). Accumulating evidence suggest that coinfected patients may present with atypical rashes, prolonged illness, or more severe systemic manifestations, although most available data come from small studies, outbreak investigations, or case reports.

Despite growing documentation, significant uncertainties persist regarding mpox coinfections in Africa. There is no comprehensive estimate of coinfection prevalence, partly due to fragmented surveillance systems and limited laboratory capacity (Hughes et al., 2021; Cheuyem et al., 2025a). Moreover, the clinical impact of specific coinfections, especially HIV, VZV, and bacterial infections, remains poorly quantified. It is unclear how coinfections modify rash morphology, duration of viral shedding, immune response, or risk of complications (Hughes et al., 2021; Cheuyem et al., 2025a). Another unresolved gap concerns interactions between viral clades, coinfection profiles and clinical manifestations (Iroezindu et al., 2023; Brosius et al., 2025). Additionally, mpox shares clinical similarities with several endemic infections, leading to common misdiagnosis. Few studies have systematically assessed diagnostic overlap (Hughes et al., 2021; Cheuyem et al., 2025a). Finally, the lack of large multicountry cohort data limits our understanding of risk factors for severe disease among coinfected individuals and constrains the development of evidence-based clinical guidelines.

Addressing these gaps is vital for both clinical practice and public health. Improved knowledge of coinfection prevalence and clinical impact would support early risk stratification, refine case definitions, and inform treatment protocols tailored to high-burden settings (Hughes et al., 2021; Cheuyem et al., 2025a). From a public-health perspective, understanding how coinfections interact with viral clades can strengthen surveillance systems, guide vaccination and testing strategies, and optimize resource allocation in low-resource contexts (Iroezindu et al., 2023; Cheuyem et al., 2025a). Such evidence is necessary to prevent severe outcomes, improve outbreak preparedness, and ensure effective and equitable control of mpox across Africa. This systematic review, therefore, aims to describe the landscape of coinfections (VZV and HIV) among confirmed mpox cases and characterize the trend of clinical presentation of clade-categorized mpox patients in Africa.

## 2 Methods

### 2.1 Study design

The Preferred Reporting Items for Systematic Review and Meta-analysis (PRISMA) guidelines were applied while reporting the study (Page et al., 2021). To ensure process rigor and transparency, the study protocol was filed in the International Prospective Register of Systematic Reviews (CRD420251133960). The final search was concluded on 27 February 2025. The protocol was registered on 26 August 2025, and revised on 27 November 2025. The data extraction was completed on 29 September 2025.

### 2.2 Eligibility criteria

Any observational studies that report on patients with mpox coinfections and clinical manifestations, conducted in Africa, were included. Populations of interest included both community-based cases and hospital-based cases, including adults and children. The key exposures of interest were coinfections occurring in mpox patients (HIV, VZV). To capture full historical and contemporary evidence, the time frame limit spanned till 2024. Only studies published in English or French were considered.

### 2.3 Exclusion criteria

Studies were excluded for the following reasons: duplication of data, focus outside the scope of our research objectives, non-observational studies (comment, letter to editor, review, other systematic review) and studies conducted outside Africa. To prevent inflating the pooled estimates inherent in the inclusion of small-sample-size studies (fewer than 10 studies), case reports and case series were excluded. We also excluded studies focusing exclusively on laboratory, genomic, or animal models without reporting clinical or coinfection data. Additionally, articles lacking full-text availability were omitted due to insufficient data or the absence of required outcome measures.

### 2.4 Article searching strategy

A comprehensive search strategy was employed to identify all relevant literature. Multiple electronic databases were searched, including PubMed, Scopus, ScienceDirect, Web of Science, CINAHL, and EMBASE. To incorporate research from African scholars, African Journals Online (AJOL) was consulted as well. In addition to these databases, we searched grey literature, which includes unpublished research and preprints. Moreover, a manual search was conducted on Google Scholar and in the reference lists of included studies. For PubMed the search strategy was a combination of search MeSH terms: (“Monkeypox” OR “Mpox” OR “Monkeypox virus” OR “MPXV” OR “Varicella-zoster virus” OR “HIV,”), AND (“Africa,” OR individual country names such as “Democratic Republic of Congo,” “Nigeria,” or “Cameroon” …) (Supplementary File 1; Supplementary Table 1). Additionally, a manual search for additional publications that were not indexed in these databases. Two study investigators (FZLC and RT) conducted the screening process and any observed discrepancies were solved through discussion or consulting a third reviewer (LBKB) to reach consensus.

### 2.5 Data extraction

We developed a Microsoft Excel 2016 form to collect study characteristics from all included study reports. This form captured the first author’s name, study year, region, study design, type of participant, setting, sampling method, total number of confirmed mpox cases, the total number VZV cases, number of VZV and mpox coinfected patients, the number of HIV and mpox coinfected patients et total number of patients tested for VZV, clade categorization, and frequency of each clinical manifestation. Two study investigators (FZLC and CA) independently extracted data. To ensure accuracy, cases of discrepancies were solved through discussion to reach consensus or by consulting a third study investigator (AEM).

### 2.6 Data quality assessment

The Joanna Briggs Institute (JBI) quality assessment tool was used to evaluate the quality of studies included (JBI Critical Appraisal Tools, 2017). This was conducted by two independent reviewers (FZLC and LBKB). Risk of bias was assessed using nine or ten criteria, depending on the study design. (1) For cross-sectional studies, criteria included: appropriateness of the sampling frame, use of a suitable sampling technique, adequate sample size, description of study subjects and setting, sufficient data analysis, use of valid methods for identifying conditions and measurements, use of appropriate statistical analysis, and an adequate response rate (≥60%). (2) For case series, criteria included: standardized measurement and valid identification of the condition for all participants, consecutive and complete inclusion of participants, reporting of participant demographics and clinical information, reporting of outcomes or follow-up results, reporting of the presenting site(s)/clinic(s) demographic information, and statistical analysis appropriate for case series studies. (3) For case reports, criteria included: clear description of patient demographic characteristics, patient history and timeline, current clinical condition on presentation, diagnostic tests/assessment methods and results, interventions/treatment procedures, post-intervention clinical condition, adverse/unanticipated events, and takeaway lessons. Each criterion was scored as 1 (yes) or 0 (no or unclear). The overall risk of bias was categorized as low (>50%), moderate (>25–50%), or high (≤25%). Any disagreements between the reviewers were resolved through discussion or by consultation with a third reviewer (AEM).

### 2.7 Outcome measurement

The primary outcomes of this systematic review and meta-analysis were the coinfection rate of mpox and VZV, or HIV. Secondary outcomes included the VZV prevalence and mpox clinical manifestation by clade classification. The mpox coinfection rate was calculated by dividing the number of confirmed VZV or HIV cases by the total number of confirmed mpox cases screened for VZV or HIV. The prevalence of VZV was calculated as the number of confirmed VZV cases divided by the total number of patients tested. For clinical features among confirmed mpox cases, the proportion of each manifestation was determined by dividing its frequency by the number of clinically assessed confirmed mpox cases.

### 2.8 Operational definition

A patient was considered laboratory-confirmed mpox if at least one specimen tested positive for Orthopoxvirus using a specific assay or Mpox-specific real-time PCR, or if mpox was isolated in culture. A case was defined as laboratory-confirmed VZV if at least one specimen yielded a positive result in a real-time PCR assay targeting the VZV-specific DNA signature (Whitehouse et al., 2021). Similarly, a case was defined as laboratory-confirmed HIV if at least one specimen showed a positive result in an HIV antigen-specific assay or HIV-specific real-time PCR (Hutin et al., 2001; Brosius et al., 2025).

### 2.9 Statistical analysis and synthesis

Study heterogeneity was assessed using the Cochrane Q statistic and the heterogeneity between studies was evaluated using the I^2^ statistic, which classified it as low (<25%), moderate (25%–75%), or high (>75%). A random-effects model was applied for all pooled analyses. To investigate potential sources of heterogeneity, we performed subgroup analyses based on study period, country, setting, and participant type. Countries were categorized according to the WHO African Region classification (ESPEN, 2023): Western (Nigeria), Eastern (Burundi and Kenya), and Central (Cameroon, Central African Republic, and DRC). The association between these study characteristics and the pooled estimates was further examined using univariable and multivariable meta-regression. We used generalized linear mixed models (GLMM) coupled with the probit-logit transformation (PLOGIT), which is robust for synthesizing proportional data, including extreme proportions of 0% or 100%, without requiring, without needing continuity corrections (Stijnen et al., 2010). Statistical significance was defined as a two-sided *p*-value <0.05. All analyses were performed using R software version 4.5.1 with the ‘meta’ package (R Core Team, 2024).

### 2.10 Publication bias and sensitivity test

Publication bias was assessed visually using a funnel plot. The asymmetry of the inverted funnel shape suggested the potential of publication bias. In addition, statistical evaluation was conducted using Egger’s linear regression test and Begg’s rank correlation test, with a *p*-value <0.05 indicating a significant risk of publication bias. The trim-and-fill method was used to adjust for potential missing studies (Shi and Lin, 2019). To assess the robustness of the findings, we performed a sensitivity analysis by iteratively excluding one study at a time and pooling the resulting estimates. We have also conducted a sensitivity analysis excluding studies with a sample size of <10 participants to assess potential influence on the pooled estimates. The GRADE (Grading of Recommendations Assessment, Development and Evaluation) was used to assess the level of certainty of our findings (Brennan and Johnston, 2023).

## 3 Results

### 3.1 Study selection

**FIGURE 1:**
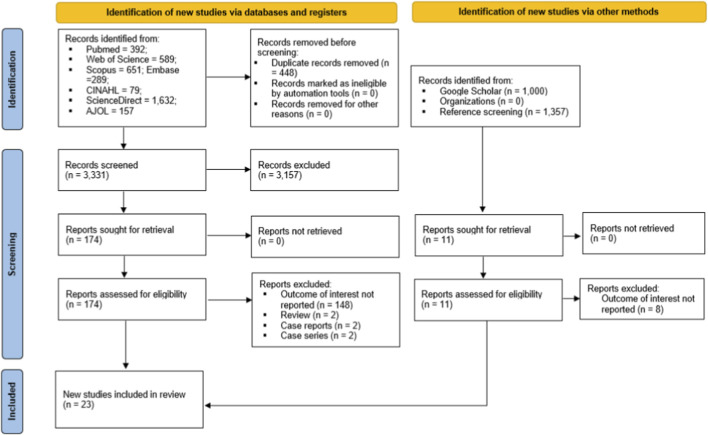
PRISMA diagram flow from study identification to inclusion in the meta-analysis (Page et al., 2021).

A total of 3,789 records were obtained from database searches, along with three additional records from other sources. After removing 448 duplicates, 3,334 articles were screened based on their titles and abstracts, resulting in the exclusion of 3,157 that did not meet our inclusion criteria. The full texts of 177 articles were then evaluated for eligibility. An additional 154 articles were excluded because they did not report the outcomes of interest (n = 148), were review articles (n = 2) or unappropriated study design (n = 4). In the end, 23 studies (including three studies from other sources) met all criteria and were included in the systematic review and meta-analysis (Figure 1).

### 3.2 Characteristics of studies included

A total of 23 studies from the Democratic Republic of the Congo (DRC; 60.9%; n = 14 studies) (Mwanba et al., 1997; Meyer et al., 2002; Nolen et al., 2016; Hoff et al., 2017; Osadebe et al., 2017; Petersen et al., 2019; Hughes et al., 2021; Whitehouse et al., 2021; Mande et al., 2022; Kinganda-Lusamaki et al., 2023; Masirika et al., 2024; Vakaniaki et al., 2024; Bangwen et al., 2025; Brosius et al., 2025), Nigeria (20.8%; n = 5 studies) (Ogoina et al., 2019; Ogoina et al., 2020; Ogoina and Yinka-Ogunleye, 2022; Stephen et al., 2022; Mmerem et al., 2024b), the Central African Republic (CAR; 4.2%; n = 1 study) (Besombes et al., 2023), Cameroon (4.2%; n = 1 study) (Djuicy et al., 2024), Kenya (4.2%; n = 1 study) (Onyango et al., 2025) and Burundi (4.2%; n = 1 study) (Nizigiyimana et al., 2024) were included. Most were cross-sectional (91.3%; n = 21 studies), community-based (73.9%; n = 17 studies) with low risk of bias (95.7%; n = 22 studies). Coinfection rates of VZV and mpox (reported in 10 studies) varied widely, from 0% in some studies to 81.8% in others. All included studies used non-probabilistic sampling (Table 1).

**TABLE 1:** Characteristics of included studies.

References	Period	Country	Design	Setting	Participant	Riks of bias	Prevalence count/Sample size (%)		
​	​	​	​	​	​	​	Mpox coinfection		​
							VZV	HIV	VZV
Aplogan et al. (1997)	1997	DRC	Cross-sectional	Community	GP	Low	NR	NR	4/19 (21.1)
Bangwen et al. (2025)	2023	DRC	Cross-sectional	Community and hospital	GP	Low	NR	NR	1,174/2,758 (42.6)
Besombes et al. (2023)	2022	CAR	Cross-sectional	Community	GP	Low	0/14 (7.1)	NR	0/42 (0.0)
Brosius et al. (2025)	2024	DRC	Cross-sectional	Hospital	GP	Moderate	NR	6/431 (1.4)	NR
Djuicy et al. (2024)	2022	Cameroon	Cross-sectional	Community and hospital	GP	Low	0/32 (0.0)	NR	0/32 (0.0)
Hoff et al. (2017)	2007	DRC	Cross-sectional	Community	GP	Low	152/783 (19.4)	NR	430/783 (54.9)
Hughes et al. (2021)	2014	DRC	Cross-sectional	Community	GP	Low	134/534 (25.1)	NR	591/1,107 (53.4)
Kinganda-Lusamaki et al. (2023)	2022	DRC	Cross-sectional	Community	GP	Low	0/154 (0.0)	NR	28/325 (8.6)
Mande et al. (2022)	2019	DRC	Cross-sectional	Community	GP	Low	NR	NR	45/56 (80.4)
Masirika et al. (2024)	2024	DRC	Cohort study	Community	GP	Low	NR	2/49 (4.1)	NR
Meyer et al. (2002)	2001	DRC	Cross-sectional	Community	GP	Low	1/14 (0.0)	NR	6/14 (42.9)
Mmerem et al. (2024b)	2023	Nigeria	Cohort study	Hospital	GP	Low	16/56 (28.6)	3/54 (5.6)	16/56 (28.6)
Nizigiyimana et al. (2024)	2024	Burundi	Cross-sectional	Community	GP	Low	NR	7/154 (4.5)	NR
Nolen et al. (2016)	2013	DRC	Cross-sectional	Community	GP and HCW	Low	NR	NR	5/60 (8.3)
Ogoina et al. (2019)	2017	Nigeria	Cross-sectional	Hospital	GP	Low	NR	3/18 (16.7)	NR
Ogoina et al. (2020)	2018	Nigeria	Cross-sectional	Hospital	GP	Low	NR	9/40 (22.5)	NR
Ogoina and Yinka-Ogunleye (2022)	2019	Nigeria	Cross-sectional	Hospital	GP	Low	NR	3/16 (18.8)	NR
Onyango et al. (2025)	2024	Kenya	Cross-sectional	Community	GP	Low	4/26 (15.4)	NR	170/277 (61.4)
Osadebe et al. (2017)	2014	DRC	Cross-sectional	Community	GP	Low	NR	NR	383/752 (50.9)
Petersen et al. (2019)	2014	DRC	Cross-sectional	Community	HCW	Low	NR	NR	3/14 (21.4)
Stephen et al. (2022)	2022	Nigeria	Cross-sectional	Hospital	GP	Low	9/11 (81.8)	NR	22/33 (66.7)
Vakaniaki et al. (2024)	2024	DRC	Cross-sectional	Community	GP	Low	NR	3/108 (2.8)	NR
Whitehouse et al. (2021)	2015	DRC	Cross-sectional	Community	GP	Low	169/1,057 (16.0)	4/1,057 (0.4)	169/1,658 (10.2)

Ref, Reference; VZV, Varicella-zoster virus; HIV, human immunodeficiency virus; DRC, Democratic Republic of Congo; CAR, Central African Republic; GP, general population; HCW, healthcare worker; NR, not reported.

### 3.3 VZV infection among confirmed mpox cases in Africa

**FIGURE 2:**
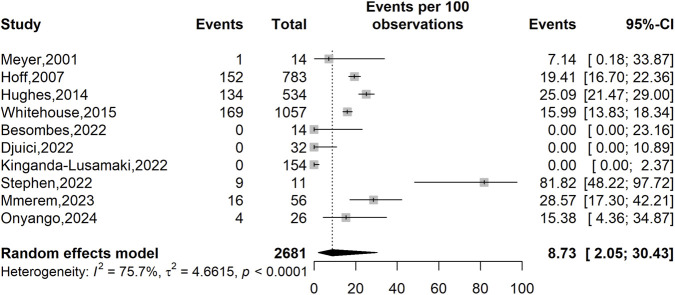
Varicella-zoster virus (VZV) infection prevalence among confirmed mpox cases in Africa.

Across 10 studies (Meyer et al., 2002; Hoff et al., 2017; Petersen et al., 2019; Hughes et al., 2021; Whitehouse et al., 2021; Stephen et al., 2022; Besombes et al., 2023; Kinganda-Lusamaki et al., 2023; Djuicy et al., 2024; Mmerem et al., 2024b; Onyango et al., 2025) (n = 2,681), the pooled prevalence of VZV–mpox coinfection was 8.73% (95% CI: 2.05–30.43). Heterogeneity was (*I* ^2^ = 75.7%, *p* < 0.001), indicating significant heterogeneity between-study differences and suggesting that the pooled estimate should be viewed with caution across different settings, populations and other methodological characteristics (Figure 2).

Subgroup analyses showed a higher pooled prevalence of VZV–mpox coinfection in studies conducted before 2022 (19.34%; 95% CI: 15.58–23.74; 2,388 participants; n = 4 studies) compared to those completed from 2022 onward (2.58%; 95% CI: 0.07–51.70; 293 participants; n = 6 studies), though this difference was not statistically significant (*p* = 0.244). A nearly significant difference was observed based on study setting (*p* < 0.058), with hospital-based studies showing much higher prevalence (53.64%; 95% CI: 16.74–86.94; 67 participants, n = 2 studies) than community-based studies (7.32%; 95% CI: 2.03–23.17; 2,582 participants; n = 7 studies). Although differences between countries were not statistically significant (*p* = 0.298), the highest pooled prevalence was observed in Nigeria (53.64%; 95% CI: 16.74–86.94; 67 participants; n = 2 studies), followed by Kenya (15.38%; 95% CI: 4.36–34.87; 26 participants, n = 1 study) and the DRC (7.92%; 95% CI: 1.70–29.93; 2,542 participants, n = 5 studies). WHO regional analysis showed a significant gradient (*p* = 0.037), with the highest prevalence in Western Africa (53.64%; 95% CI: 16.74–86.94; 68 participants, n = 2 studies) and the lowest in Central Africa (3.60%; 95% CI: 0.51–21.49; 2,588 participants; n = 7 studies) (Table 2).

**TABLE 2:** Subgroup meta-analysis of the pooled prevalence of varicella-zoster virus infection among confirmed mpox cases in Africa.

Subgroup	Sample size	Prevalence^1^ (%)	95% CI limits^1^		Number of reports	Heterogeneity statistic^1^		Subgroup difference
			Lower	Upper		*I* ^2^ (%)	*p*-value	*p*-value
Study period (year)	​	​	​	​	​	​	​	0.245
Before 2022	2,388	19.34	15.58	23.74	4	85.0	<0.001	​
2022 +	293	2.93	0.07	51.70	6	56.9	0.041	​
Study design	​	​	​	​	​	​	​	0.078
Cross-sectional	2,625	6.89	1.23	30.55	9	76.7	<0.001	​
Cohort	56	28.57	17.30	42.21	1	—	—	​
Study setting	​	​	​	​	​	​	​	0.058
Community	2,582	7.32	2.03	23.17	7	70.4	0.003	​
Community and hospital	32	0.00	0.00	10.89	1	—	—	​
Hospital	68	60.66	19.46	90.78	3	76.1	0.015	​
Country	​	​	​	​	​	​	​	0.298
DRC	2,542	7.92	1.70	29.93	5	80.0	<0.001	​
CAR	14	0.00	0.00	23.16	1	—	—	​
Cameroon	32	0.00	0.00	10.89	1	—	—	​
Nigeria	67	53.64	16.74	86.94	2	88.1	0.004	​
Kenya	26	15.38	4.36	34.87	1	—	—	​
WHO zone^2^	​	​	​	​	​	​	​	0.037
Western	67	53.64	16.74	86.94	2	88.1	0.004	​
Eastern	26	15.38	4.36	34.87	1	—	—	​
Central	2,588	3.60	0.51	21.49	7	70.0	0.003	​

1 Random effects model; CI: confidence interval; WHO: World Health Organization.

2 Western: Nigeria; Eastern: Kenya; Central: Cameroon, CAR, Central African Republic, and DRC, Democratic Republic of Congo.

### 3.4 HIV infection among confirmed mpox cases in Africa

**FIGURE 3:**
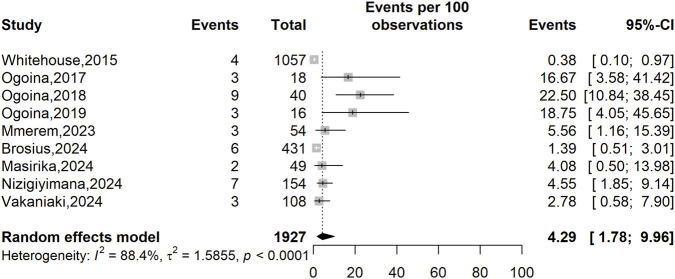
Human immunodeficiency virus (HIV) infection prevalence among confirmed mpox cases in Africa.

Across 9 studies (Ogoina et al., 2019; Ogoina et al., 2020; Whitehouse et al., 2021; Ogoina and Yinka-Ogunleye, 2022; Masirika et al., 2024; Mmerem et al., 2024b; Nizigiyimana et al., 2024; Vakaniaki et al., 2024; Brosius et al., 2025) involving 1,939 confirmed mpox cases, the pooled prevalence of HIV coinfection was 4.29% (95% CI: 1.78–9.96; *I* ^2^ = 88.4%, *p* < 0.0001). Heterogeneity was high, indicating substantial difference between studies and suggesting that the pooled estimate should be interpreted with caution (Figure 3).

Subgroup analyses showed lower pooled prevalence of HIV coinfection in studies conducted from 2022 onward (2.87%; 95% CI: 1.65–4.98; 796 participants, n = 5 studies) compared with studies conducted before 2022 (6.98%; 95% CI: 1.21–31.45; 1,131 participants, n = 4 studies), although this difference was not statistically significant (*p* = 0.337). A significant difference was observed by study setting (*p* = 0.044), with hospital-based studies reporting higher prevalence (8.24%; 95% CI: 2.98–20.83; 559 participants; n = 5 studies) than community-based studies (1.83%; 95% CI: 0.63–5.23; 1,368 participants; n = 4 studies). Country-level differences were statistically significant (*p* < 0.001), with higher prevalence of HIV coinfection in Nigeria (14.06%; 95% CI: 7.67–24.35; 128 participants; n = 4 studies) than in the DRC (1.24%; 95% CI: 0.49–3.07; 1,645 participants; n = 4 studies). WHO regional analysis mirrored country-level findings (*p* < 0.001), with the highest prevalence in the Western WHO zone (14.06%; 95% CI: 7.67–24.35; 128 participants; n = 4 studies) and the lowest in Central zone (1.24%; 95% CI: 0.49–3.07; 1,645 participants; n = 4 studies) (Table 3).

**TABLE 3:** Subgroup meta-analysis of the pooled prevalence of human immunodeficiency virus (HIV) infection among confirmed mpox cases in Africa.

Subgroup	Sample size	Prevalence^1^ (%)	95% CI limits^1^		Number of reports	Heterogeneity statistic^1^		Subgroup difference
			Lower	Upper		*I* ^2^ (%)	*p*-value	*p*-value
Study period (year)	​	​	​	​	​	​	​	0.337
Before 2022	1,131	6.98	1.21	31.45	4	94.4	<0.001	​
2022 +	796	2.87	1.65	4.98	5	36.1	0.181	​
Study design	​	​	​	​	​	​	​	0.878
Cross-sectional	1,824	4.36	1.44	12.40	7	91.3	<0.001	​
Cohort	103	4.85	2.03	11.14	2	0.0	0.729	​
Study setting	​	​	​	​	​	​	​	0.044
Community	1,368	1.83	0.63	5.23	4	82.7	<0.001	​
Hospital	559	8.24	2.98	20.83	5	88.3	<0.001	​
Country	​	​	​	​	​	​	​	<0.001
DRC	1,645	1.24	0.49	3.07	4	71.6	0.014	​
Nigeria	128	14.06	7.67	24.35	6	42.8	0.155	​
Burundi	154	4.55	1.85	9.14	1	—	—	​
WHO zone^2^	​	​	​	​	​	​	​	<0.001
Western	128	14.06	7.67	24.35	4	42.8	0.155	​
Eastern	154	4.55	1.85	9.14	1	—	—	​
Central	1,645	1.24	0.49	3.07	4	71.6	0.014	​

1 Random effects model; CI: confidence interval; WHO: World Health Organization.

2 Western: Nigeria; Eastern: Kenya; Central: Cameroon, CAR, Central African Republic, and DRC, Democratic Republic of Congo.

### 3.5 Varicella-zoster virus infections in Africa

**FIGURE 4:**
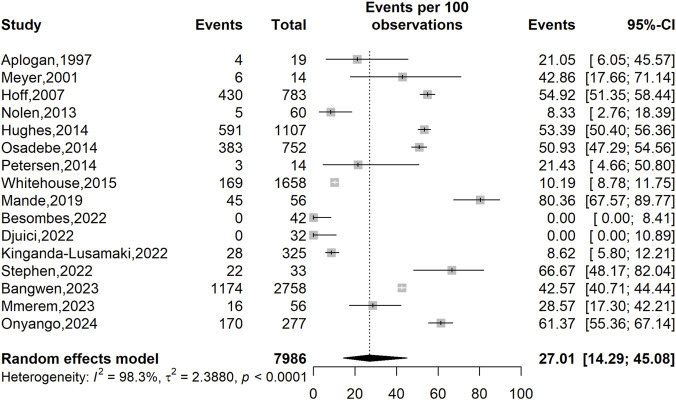
Prevalence of varicella-zoster virus (VZV) infections in Africa.

Across 16 studies involving 7,986 participants, the overall prevalence of varicella-zoster virus (VZV) infection in Africa was 27.01% (95% CI: 14.29–45.08; *I* ^2^ = 98.3%, *p* < 0.001). The high heterogeneity suggested that the pooled prevalence estimate should be interpreted with caution (Figure 4).

Subgroup analyses indicated higher pooled VZV prevalence in studies conducted before 2022 (35.02%; 95% CI: 19.52–54.50; 4,463 participants; n = 9 studies) compared to those published from 2022 onward (15.42%; 95% CI: 2.89–52.72; 3,523 participants; n = 7 studies) although this difference was not statistically significant (*p* = 0.283). In contrast, notable differences were observed by countries (*p* = 0.033) and WHO region (*p* = 0.002). The highest prevalence was reported in Kenya (61.37%; 95% CI: 55.36–67.14; 277 participants; n = 1 study) and Nigeria (46.49%; 95% CI: 21.83–73.00; 89 participants; n = 2 studies), while lower prevalence estimates were seen in the Democratic Republic of Congo (31.96%; 95% CI: 18.56–49.18; 7,546 participants; n = 11 studies) and across Central Africa overall (21.80%; 95% CI: 9.77–41.80; 7,620 participants; n = 13 studies) (Table 4).

**TABLE 4:** Subgroup meta-analysis of the pooled prevalence of varicella-zoster virus infections in Africa.

Subgroup	Sample size	Prevalence^1^ (%)	95% CI limits^1^		Number of reports	Heterogeneity statistic^1^		Subgroup difference
			Lower	Upper		*I* ^2^ (%)	*p*-value	*p*-value
Study period (year)	​	​	​	​	​	​	​	0.283
Before 2022	4,463	35.02	19.52	54.50	9	98.9	<0.001	​
2022 +	3,523	15.42	2.89	52.72	7	96.2	<0.001	​
Study design	​	​	​	​	​	​	​	0.858
Cross-sectional	7,930	26.67	13.29	46.32	15	98.4	<0.001	​
Cohort	56	28.57	17.30	42.21	1	—	—	​
Study setting	​	​	​	​	​	​	​	0.366
Community	5,107	27.65	13.71	47.91	12	98.7	<0.001	​
Hospital	89	46.49	21.83	73.00	2	91.4	<0.001	​
Community and hospital	2,790	4.28	0.02	91.54	2	0.0	0.999	​
Participant	​	​	​	​	​	​	​	​
General population	7,912	29.47	14.76	50.19	14	98.5	<0.001	​
Healthcare worker	14	21.43	4.66	50.80	1	—	—	​
General population and healthcare worker	60	8.33	2.76	18.39	1	—	—	​
Country	​	​	​	​	​	​	​	0.033
DRC	7,546	31.96	18.56	49.18	11	98.8	<0.001	​
Nigeria	89	46.49	21.83	73.00	2	91.4	0.007	​
CAR	42	0.00	0.00	8.41	1	—	—	​
Cameroon	32	0.00	0.00	10.89	1	—	—	​
Kenya	277	61.37	55.36	67.14	1	—	—	​
WHO zone^2^	​	​	​	​	​	​	​	0.002
Western	89	46.49	21.83	73.00	2	91.4	<0.001	​
Eastern	277	61.37	55.36	67.14	1	—	—	​
Central	7,620	21.80	9.77	41.80	13	98.5	<0.001	​

1 Random effects model; CI: confidence interval; WHO: World Health Organization.

2 Western: Nigeria; Eastern: Kenya; Central: Cameroon, CAR, central african republic, and DRC, democratic republic of congo.

### 3.6 Clinical pattern of confirmed mpox cases by genotypic category in Africa

Clinical manifestations of mpox varied significantly by viral clade. Rash was nearly universal across clades (>96%) and did not differ by genotype (*p* = 0.118). In contrast, systemic symptoms (fever, lymphadenopathy, sore throat, myalgia, fatigue, headache, and chills) were significantly more frequent in clades I and Ia than in clade II (all *p* < 0.01). Fever and lymphadenopathy were almost universal in clades I/Ia (≥90–100%) but occurred less frequently in clade II, particularly fever (16.8%).

Mucocutaneous manifestations also differed by clade, with oral lesions, conjunctivitis, palm and sole lesions, and genital lesions occurring more often in clades I and Ia (p < 0.001 for most). Gastrointestinal and respiratory symptoms showed no significant clade-specific differences (p > 0.05). Overall, clades I and Ia were associated with more pronounced systemic disease, whereas clade II showed a milder systemic profile. Similarly, the Congo Basin clade case significantly revealed a richer clinical pattern than the West Africa clade (Table 5; Supplementary File 4; Supplementary Table 1).

**TABLE 5:** Descriptive narrative synthesis of the clinical profile of confirmed mpox cases by geographical clade classification in Africa.

Symptoms	Geographical clade categorization				p-value
	Congo basin		West Africa		
	Total	*n* (%)	Total	*n* (%)	
Rash	3,458	3,447 (99.7)	4	4 (100.0)	1.000
Fever	2,691	2,668 (99.1)	96	84 (87.5)	<0.001***
Lymphadenopathy	2,783	2,721 (97.8)	69	48 (69.6)	<0.001***
Headache	2,174	1,482 (68.2)	81	62 (76.5)	0.091
Pruritus or itchy lesion	1,397	1,193 (85.4)	78	57 (73.1)	0.003**
Sore throat or dysphagia	2,730	2,313 (84.7)	77	45 (58.4)	<0.001***
Palm lesions	1,382	1,177 (85.2)	74	49 (66.2)	<0.001***
Fatigue	2,175	1,671 (76.8)	118	61 (51.7)	<0.001***
Chills or sweat	1,704	1,329 (78.0)	118	74 (62.7)	<0.001***
Sole lesions	1,289	990 (76.8)	66	42 (63.6)	0.016*
Oral lesions	2,099	1,541 (73.4)	118	43 (36.4)	<0.001***
Myalgia	1,737	1,194 (68.7)	67	42 (62.7)	0.308
Genital lesions	716	508 (70.9)	69	45 (65.2)	0.359
Malaise	963	732 (76.0)	4	1 (25.0)	0.022*
Anorexia	1,288	679 (52.7)	4	1 (25.0)	0.313
Cough	3,213	1,677 (52.2)	118	33 (28.0)	<0.001***
Conjunctivitis	2,754	527 (19.1)	118	27 (22.9)	0.317
Vomiting or nausea	2,325	555 (23.9)	118	26 (22.0)	0.659
Light sensitivity	1,331	445 (33.4)	118	26 (22.0)	0.011*
Painful lesion	426	333 (78.2)	—	—	—
Parotiditis	3	2 (66.7)	—	—	—
None	12	8 (66.7)	—	—	—
Anal lesions	431	136 (31.5)	—	—	—
Rhinorrhea	216	67 (31.0)	—	—	—
Abdominal pain	1,283	391 (30.5)	—	—	—
Bedridden status	2,739	707 (25.8)	—	—	—
Joint pain	849	189 (22.3)	—	—	—
Painful eye	426	85 (20.0)	—	—	—
Back pain	216	25 (11.6)	—	—	—
Dyspnea	946	111 (11.7)	—	—	—
Diarrhea	1,386	159 (11.5)	—	—	—
Rectal pain	424	46 (10.8)	—	—	—
Facial oedema	10	1 (10.0)	—	—	—
Keloids	633	49 (7.7)	—	—	—
Ear pain	216	15 (6.9)	—	—	—
Corneal lesions	303	20 (6.6)	—	—	—
Visual problem	1,275	75 (5.9)	—	—	—
Chest pain	216	11 (5.1)	—	—	—
Hematuria	428	22 (5.1)	—	—	—
Eye lesion	633	28 (4.4)	—	—	—
Neck stiffness	216	9 (4.2)	—	—	—
Confusion	1,278	52 (4.1)	—	—	—
Hemorrhagic skin lesions	225	8 (3.6%)	—	—	—
Dehydration	216	7 (3.2%)	—	—	—
Hypothermia	226	4 (1.8%)	—	—	—
Convulsions	1,062	3 (0.3%)	—	—	—
Alopecia	633	6 (0.9%)	—	—	—
Petechiae	216	2 (0.9%)	—	—	—
Earing problem	216	1 (0.5%)	—	—	—
Oedema	216	1 (0.5%)	—	—	—
Number of studies	18	​	6	​	​
Country [References]	DRC, CAR, Congo and Sudan (Jezek et al., 1987; Boumandouki et al., 2007; Formenty et al., 2010; Kalthan et al., 2016 ; 2018; Hoff et al., 2017; Nakoune et al., 2017; Osadebe et al., 2017; Besombes et al., 2019 ; 2023; Hughes et al., 2021; Whitehouse et al., 2021; Mande et al., 2022; Pittman et al., 2023; Mukadi-Bamuleka et al., 2024; Vakaniaki et al., 2024; Brosius et al., 2025)	​	Nigeria ( Ogoina et al., 2019; Ogoina et al., 2020; Yinka-Ogunleye et al., 2019; Echekwube et al., 2020; Mmerem et al., 2024a; Mmerem et al., 2024b )	​	​

1 Chi square or Fisher exact test; CAR: Central African Republic; DRC: Democratic Republic of Congo.

*** p < 0.001.

** p < 0.01.

* p < 0.05.

### 3.7 Publication bias, sensitivity analysis and GRADE assessment

Although the observed asymmetry of funnel plots for all the three outcomes assessed in this study, there was no statistically significant evidence of publication bias after performing the Egger’s (*p* = 0.689; *p* = 0.493) and Begg’s (*p* = 0.421; *p* = 0.071) tests. In addition, the trim-and-fill analysis did not impute any missing study in the pooled HIV prevalence among confirmed mpox cases (Supplementary File 1; Supplementary Figure 6; Supplementary File 2; Supplementary Figures 6, 7; Supplementary File 3; Supplementary Figure 7).

The sensitivity analysis using the leave-one-out method showed that no single study significantly influenced the three pooled estimates. These observations confirmed the robustness of the pooled estimates (Supplementary File 1; Supplementary Figure 7; Supplementary File 2; Supplementary Figure 8; Supplementary File 3; Supplementary Figure 8).

The level of certainty (GRADE) of our findings was considered low due to the type of primary studies included (observational studies), imprecision (wide confidence intervals), and inconsistency (high heterogeneity across included reports) (Supplementary File 4; Supplementary Table 2).

## 4 Discussion

This systematic review and meta-analysis aimed to determine the coinfection rate of mpox and VZV, and the clinical profile of mpox cases in Africa. It compiled data from 23 studies across Africa. Our meta-analytic approach adds substantial value beyond a traditional narrative review by quantitatively synthesizing heterogeneous data from 23 studies across Africa, thereby resolving inconsistencies in reported coinfection rates and revealing robust patterns such as the significantly higher prevalence of HIV–mpox coinfection in hospital settings that would not be apparent from any single study alone. By generating these actionable, continent-specific estimates, our work provides evidence-based guidance for targeted screening, risk stratification, and integrated coinfection management, thereby directly informing surveillance and clinical practice in endemic African settings.

### 4.1 VZV-mpox coinfection

Our meta-analysis of 2,681 confirmed mpox cases in Africa showed a pooled VZV coinfection rate of 9%. This indicates that VZV coinfection is a common feature of mpox epidemiology, especially in endemic areas and during outbreaks (Hoff et al., 2017; Stephen et al., 2022). Overall, these findings showed significant variability in estimates across different studies (*I* ^2^ = 75.7%) and should be interpreted with caution, possibly due to differences in study settings, study designs, and study populations.

The subgroup analysis revealed a significantly higher prevalence (*p* = 0.015) in hospital-based studies (61%) compared to community-based studies (7%). This difference may be attributed to the fact that patients admitted for mpox and coinfected with VZV might present with more severe disease or atypical clinical presentations, which lead the patient to seek hospital care (Benites-Zapata et al., 2022). Furthermore, the clinical similarities between VZV and mpox skin lesions may lead to misclassification, potentially affecting prevalence estimates in a hospital setting (Yinka-Ogunleye et al., 2019; Bourner et al., 2024).

Regarding the regional distribution of VZV–mpox coinfection, the highest prevalence was reported in West Africa (61%) compared to other regions. The main mpox hotspots in these regions are Nigeria, Uganda, and the DRC, respectively, which together account for more than 90% of mpox-related deaths in Africa (WHO, 2022; Nigeria CDC, 2025; Cheuyem et al., 2026). This regional variation may be due to differences in surveillance systems and diagnostic capacities. Nigeria and Uganda have improved their surveillance and diagnostic capabilities, which help identify and report cases (Boboye, 2019; WHO, 2024b). In contrast, despite being an endemic focus, the DRC faces significant surveillance limitations, especially in remote regions, leading to underreporting of coinfected cases (Adigun et al., 2024). Furthermore, differences in access to diagnostic tools, especially PCR testing, directly affect the reported prevalence estimates across regions (Bourner et al., 2024).

### 4.2 HIV-mpox coinfection

The overall prevalence of HIV coinfection among patients with mpox in our analysis was 4%. Indeed, several studies have confirmed the presence of HIV–mpox coinfection, especially in Nigeria and the DRC (Echekwube et al., 2020; Mmerem et al., 2024b; Cheuyem et al., 2025a). This implies that HIV–mpox coinfection does occur and may reach variable proportions depending on the context. The high heterogeneity observed suggests that we should interpret the pooled estimate with caution.

The WHO regional analysis mirrors the country-level findings, with the highest prevalence significantly observed (*p* < 0.001) in the West Africa region (14%) compared to other regions. In contrast, a recent meta-analysis examining the geographic and temporal variation of mpox patients living with HIV worldwide reported substantially higher HIV–mpox coinfection prevalences among mpox cases in Europe (41%) and North America (52%) (Gandhi et al., 2023). These findings highlight significant geographic differences in HIV–mpox coinfection patterns and suggest that screening and management strategies should be customized to the local epidemiological context.

At the country level, a higher prevalence was observed in Nigeria (14%), an intermediate prevalence in Burundi (5%), and a lower prevalence in the DRC (1%). This variation may be explained by several factors, including the decreasing gradient of HIV prevalence across these countries (Nigeria > Burundi > DRC), which mechanically influences the risk of coinfection (World Bank Open Data, 2025). Additionally, mpox epidemiological profiles vary greatly across different settings. In the DRC, mpox transmission has mainly been driven by zoonotic and intrafamilial contacts, mostly impacting rural populations and children, among whom HIV rates are low. Conversely, recent outbreaks in Nigeria have involved ongoing human-to-human transmission, including sexual contact, which overlaps with HIV transmission networks (McCollum et al., 2015; Ogoina et al., 2020).

A significant difference was also observed according to study setting (*p* = 0.044), with hospital-based studies reporting a substantially higher prevalence of HIV–mpox co-infection (8%) compared with community-based studies (2%). These findings are consistent with a recent meta-analysis showing that individuals co-infected with HIV and mpox had a significantly higher likelihood of hospitalization than those infected with mpox alone (OR = 1.85) (Taha et al., 2024). Similarly, another meta-analysis reported a pooled 56.6% increased risk of hospitalization among HIV-positive mpox cases compared with HIV-negative individuals (95% CI: 18.0%–107.7%) (Shabil et al., 2025). These findings support the biological plausibility that people living with HIV, due to immune system impairment, are more susceptible to developing severe or complicated forms of mpox, which increases the likelihood of hospital-based case detection (Echekwube et al., 2020; Mmerem et al., 2024b).

The marked disparity in coinfection rates between hospital-based and community-based settings suggests that surveillance strategies should prioritize targeted screening in hospitalized mpox patients to ensure efficient use of resources. The significantly higher hospitalization risk associated with HIV coinfection enables risk stratification. Patients with known HIV infection presenting with mpox should be therefore managed as potentially severe cases, with closer monitoring and prompt supportive care. Finally, the overlapping clinical features between mpox and VZV, combined with documented coinfections, argue for integrated management of coinfections, including routine HIV and VZV testing for all confirmed mpox cases, especially in hospital settings. Adopting such integrated approaches would strengthen outbreak preparedness and improve patient outcomes across the continent.

### 4.3 Mpox clinical presentation

In this study, skin rash was the predominant clinical manifestation, observed in more than 96% of cases, which is consistent with the World Health Organization (WHO) case definitions that consider rash to be the cardinal symptom of suspected mpox (WHO, 2024a). This finding confirms the robustness of current clinical criteria for case identification, particularly in resource-limited settings.

Beyond cutaneous manifestations, we observed a high frequency of systemic symptoms, including fever, lymphadenopathy, sore throat, myalgia, fatigue, headache, and chills, with a significantly higher prevalence in clades I and Ia compared with clade II. This distribution is consistent with historical data suggesting a more severe clinical presentation associated with clade I, characterized by marked systemic involvement. Indeed, large clinical series and outbreak investigations conducted in Africa have reported a high prevalence of generalized symptoms among patients infected with this clade, supporting the hypothesis of intrinsically higher virulence compared with clade II (Hutin et al., 2001; Besombes et al., 2023; Djuicy et al., 2024).

Our supporting evidence is strengthened by enhanced surveillance studies and analyses of previous outbreaks, which show that fever, chills, severe fatigue, headache, and lymphadenopathy often accompany infections attributed to clades I and Ia (Formenty et al., 2010; Stephen et al., 2022). However, the predominance of these non-specific symptoms complicates the differential diagnosis, particularly in endemic regions where febrile illnesses such as malaria, varicella-zoster virus (VZV) infection, and other viral or bacterial infections are highly prevalent (Viral Febrile Illnesses and Emerging Pathogens, 2020; Africa CDC, 2025). This clinical overlap might lead to delays in diagnosis or missed mpox cases, especially when the rash is absent or appears late.

Despite the overall high quality of the included research, significant heterogeneity remained across several analyses, highlighting significant gaps in surveillance coverage, diagnostic capabilities, and study design throughout the continent. In addition, some estimates were based on few studies with wide confidence intervals; therefore, these subgroup findings should be interpreted cautiously. The restriction to French or English articles might have limited the number of primary studies included. The fact that not all African regions were represented in the analysis might limit the generalizability of study findings. While our pooled estimates provided the best available synthesis of current evidence, the low certainty suggested findings should be interpreted with caution.

In conclusion, coinfection with VZV and HIV among mpox patients in Africa has been documented and may be associated with more severe disease and atypical or pronounced clinical presentations. The significantly higher prevalence of coinfections observed in hospital settings suggests that these coinfections may contribute to disease severity, necessitating specialized care. Clinical manifestations varied considerably by viral clade, with clades I and Ia associated with greater systemic involvement than clade II, highlighting the need to update clinical guidelines to reflect clade-specific features. Based on this hypothesis-generating evidence, further research is needed to determine whether targeted screening in hospital settings, strengthening of diagnostic networks, and enhanced genomic characterization of circulating strains could improve patient management and outbreak preparedness in Africa. These potential strategies, may be feasible through strategic resource allocation and alignment with existing disease surveillance platforms. However, to enable formal policy recommendations, prospective studies with standardized sampling and higher-certainty designs are needed to confirm these observations.

## Data Availability

The original contributions presented in the study are included in the article/Supplementary Material, further inquiries can be directed to the corresponding author.
