# Supplementary material for: Mpox coinfections and clinical manifestation in Africa: a systematic review and meta-analysis
Source: Front Syst Biol. 2026 May 7;6:1795422. doi: 10.3389/fsysb.2026.1795422 (PMC13189820; doi:10.3389/fsysb.2026.1795422)
Supplement: Supplementary file 1 [file Supplementaryfile1.docx]

**Supplementary Table 1** Searching strategy and results by database

| **Database** | **Results by February 2025** |
| --- | --- |
| **PubMed:**  (Monkeypox OR mpox OR monkeypox)  AND  (“Varicella-zoster virus” OR HIV)  AND  (Africa OR African OR Algeria OR Angola OR Benin OR Botswana OR "Burkina Faso" OR Burundi OR Cameroon OR "Cape Verde" OR "Central African Republic" OR Chad OR Comoros OR Congo OR "Democratic Republic of the Congo" OR Djibouti OR Egypt OR "Equatorial Guinea" OR Eritrea OR Eswatini OR Ethiopia OR Gabon OR Gambia OR Ghana OR Guinea OR "Guinea-Bissau" OR "Ivory Coast" OR Kenya OR Lesotho OR Liberia OR Libya OR Madagascar OR Malawi OR Mali OR Mauritania OR Mauritius OR Morocco OR Mozambique OR Namibia OR Niger OR Nigeria OR Rwanda OR "Sao Tome and Principe" OR Senegal OR Seychelles OR "Sierra Leone" OR Somalia OR "South Africa" OR "South Sudan" OR Sudan OR Tanzania OR Togo OR Tunisia OR Uganda OR Zambia OR Zimbabwe) | 392 |
| **Web of Science:**  Monkeypox OR mpox OR monkeypox  AND  (“Varicella-zoster virus” OR HIV)  AND  (Africa OR African OR Algeria OR Angola OR Benin OR Botswana OR "Burkina Faso" OR Burundi OR Cameroon OR "Cape Verde" OR "Central African Republic" OR Chad OR Comoros OR Congo OR "Democratic Republic of the Congo" OR Djibouti OR Egypt OR "Equatorial Guinea" OR Eritrea OR Eswatini OR Ethiopia OR Gabon OR Gambia OR Ghana OR Guinea OR "Guinea-Bissau" OR "Ivory Coast" OR Kenya OR Lesotho OR Liberia OR Libya OR Madagascar OR Malawi OR Mali OR Mauritania OR Mauritius OR Morocco OR Mozambique OR Namibia OR Niger OR Nigeria OR Rwanda OR "Sao Tome and Principe" OR Senegal OR Seychelles OR "Sierra Leone" OR Somalia OR "South Africa" OR "South Sudan" OR Sudan OR Tanzania OR Togo OR Tunisia OR Uganda OR Zambia OR Zimbabwe) | 589 |
| **Scopus:**  (Monkeypox OR mpox OR monkeypox)  AND  (“Varicella-zoster virus” OR HIV)  AND  (vaccine OR vaccination OR "vaccine acceptance" OR "vaccine uptake" OR "vaccine coverage" OR "vaccine hesitancy"  AND  Africa OR African OR Algeria OR Angola OR Benin OR Botswana OR "Burkina Faso" OR Burundi OR Cameroon OR "Cape Verde" OR "Central African Republic" OR Chad OR Comoros OR Congo OR "Democratic Republic of the Congo" OR Djibouti OR Egypt OR "Equatorial Guinea" OR Eritrea OR Eswatini OR Ethiopia OR Gabon OR Gambia OR Ghana OR Guinea OR "Guinea-Bissau" OR "Ivory Coast" OR Kenya OR Lesotho OR Liberia OR Libya OR Madagascar OR Malawi OR Mali OR Mauritania OR Mauritius OR Morocco OR Mozambique OR Namibia OR Niger OR Nigeria OR Rwanda OR "Sao Tome and Principe" OR Senegal OR Seychelles OR "Sierra Leone" OR Somalia OR "South Africa" OR "South Sudan" OR Sudan OR Tanzania OR Togo OR Tunisia OR Uganda OR Zambia OR Zimbabwe ) | 651 |
| **CINAHL:**  (Monkeypox OR mpox OR monkeypox)  AND  (“Varicella-zoster virus” OR HIV)  AND  (vaccine OR vaccination OR "vaccine acceptance" OR "vaccine uptake" OR "vaccine coverage" OR "vaccine hesitancy"  AND  (Africa OR African OR Algeria OR Angola OR Benin OR Botswana OR "Burkina Faso" OR Burundi OR Cameroon OR "Cape Verde" OR "Central African Republic" OR Chad OR Comoros OR Congo OR "Democratic Republic of the Congo" OR Djibouti OR Egypt OR "Equatorial Guinea" OR Eritrea OR Eswatini OR Ethiopia OR Gabon OR Gambia OR Ghana OR Guinea OR "Guinea-Bissau" OR "Ivory Coast" OR Kenya OR Lesotho OR Liberia OR Libya OR Madagascar OR Malawi OR Mali OR Mauritania OR Mauritius OR Morocco OR Mozambique OR Namibia OR Niger OR Nigeria OR Rwanda OR "Sao Tome and Principe" OR Senegal OR Seychelles OR "Sierra Leone" OR Somalia OR "South Africa" OR "South Sudan" OR Sudan OR Tanzania OR Togo OR Tunisia OR Uganda OR Zambia OR Zimbabwe) | 79 |
| **Embase:**  (Monkeypox OR mpox OR monkeypox)  AND  (“Varicella-zoster virus” OR HIV)  AND  (Africa OR African OR Algeria OR Angola OR Benin OR Botswana OR "Burkina Faso" OR Burundi OR Cameroon OR "Cape Verde" OR "Central African Republic" OR Chad OR Comoros OR Congo OR "Democratic Republic of the Congo" OR Djibouti OR Egypt OR "Equatorial Guinea" OR Eritrea OR Eswatini OR Ethiopia OR Gabon OR Gambia OR Ghana OR Guinea OR "Guinea-Bissau" OR "Ivory Coast" OR Kenya OR Lesotho OR Liberia OR Libya OR Madagascar OR Malawi OR Mali OR Mauritania OR Mauritius OR Morocco OR Mozambique OR Namibia OR Niger OR Nigeria OR Rwanda OR "Sao Tome and Principe" OR Senegal OR Seychelles OR "Sierra Leone" OR Somalia OR "South Africa" OR "South Sudan" OR Sudan OR Tanzania OR Togo OR Tunisia OR Uganda OR Zambia OR Zimbabwe) | 289 |
| **ScienceDirect:**  (Monkeypox OR mpox OR monkeypox)  AND  (“Varicella-zoster virus” OR HIV)  AND  (Africa OR African OR Algeria OR Angola OR Benin OR Botswana OR "Burkina Faso" OR Burundi OR Cameroon OR "Cape Verde" OR "Central African Republic" OR Chad OR Comoros OR Congo OR "Democratic Republic of the Congo" OR Djibouti OR Egypt OR "Equatorial Guinea" OR Eritrea OR Eswatini OR Ethiopia OR Gabon OR Gambia OR Ghana OR Guinea OR "Guinea-Bissau" OR "Ivory Coast" OR Kenya OR Lesotho OR Liberia OR Libya OR Madagascar OR Malawi OR Mali OR Mauritania OR Mauritius OR Morocco OR Mozambique OR Namibia OR Niger OR Nigeria OR Rwanda OR "Sao Tome and Principe" OR Senegal OR Seychelles OR "Sierra Leone" OR Somalia OR "South Africa" OR "South Sudan" OR Sudan OR Tanzania OR Togo OR Tunisia OR Uganda OR Zambia OR Zimbabwe)  To comply with the database recommendations, no more than 8 Boolean connector were used for each search | 1,632 |
| **African Journals Online (AJOL):**  (Monkeypox OR mpox OR monkeypox)  AND  (“Varicella-zoster virus” OR HIV) | 157 |

**Study period**


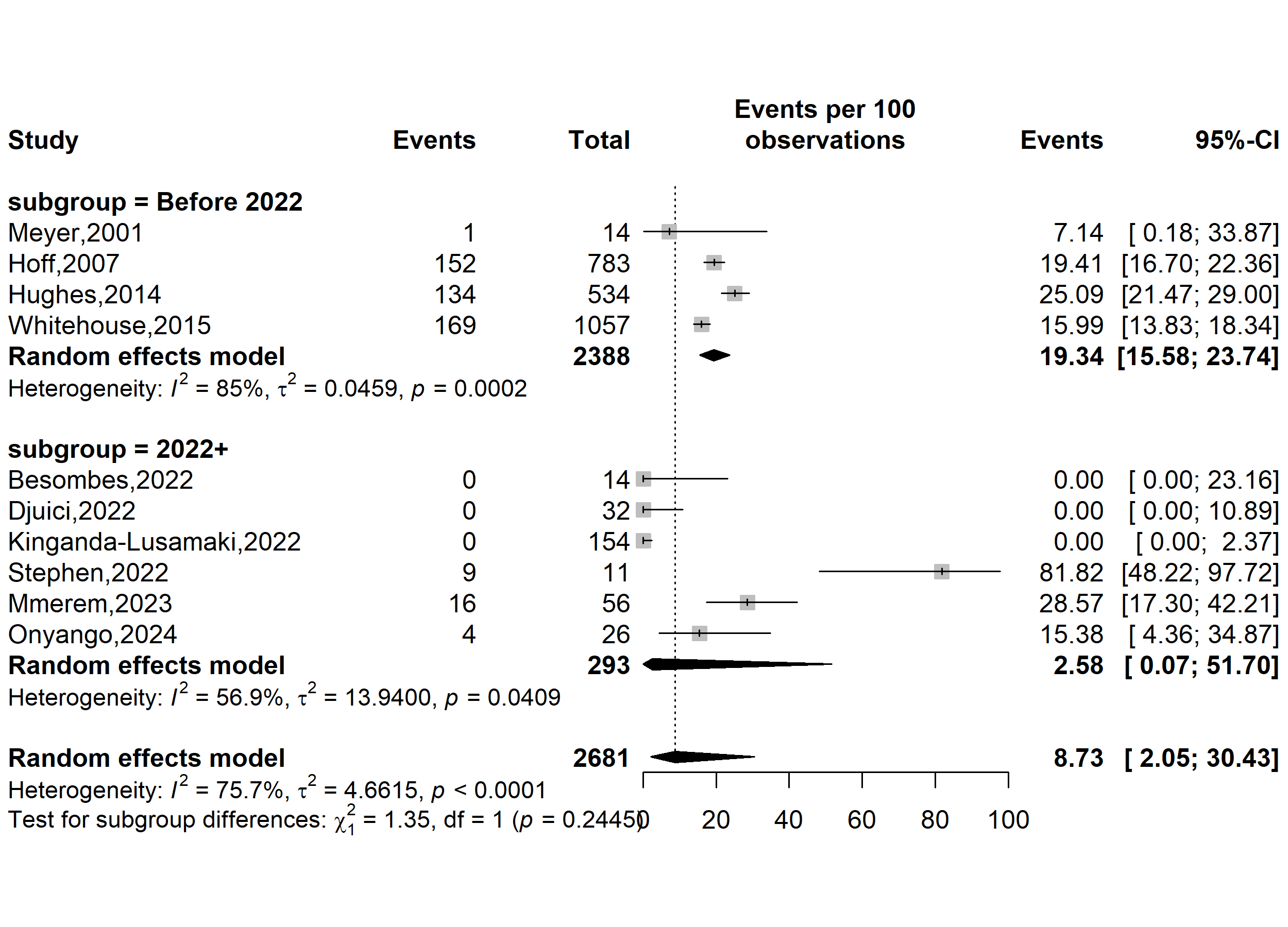


**Supplementary Fig. 1** Prevalence varicella-zoster virus (VZV) coinfections among confirmed mpox cases in Africa by study period

**Country**


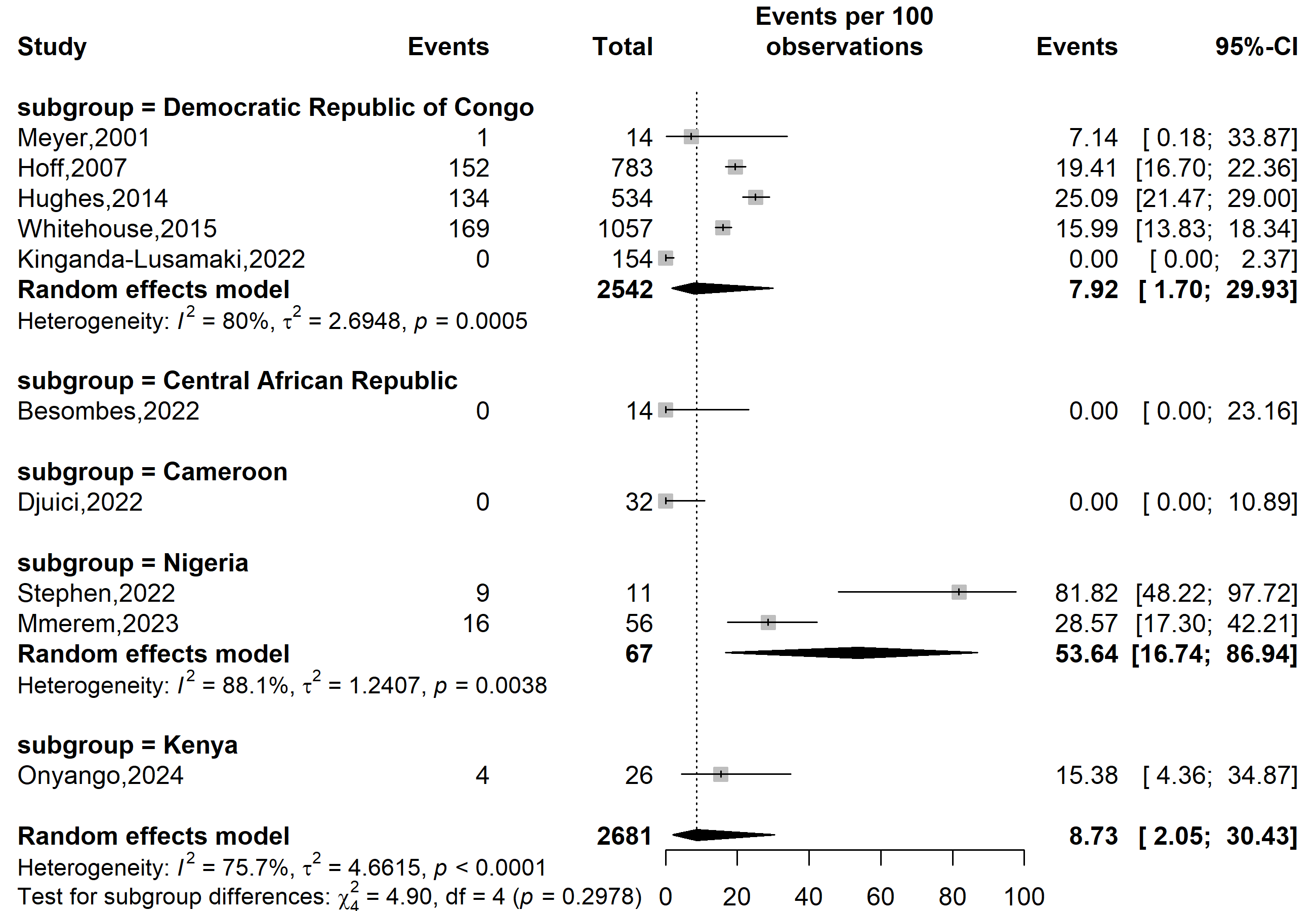


**Supplementary Fig. 2** Prevalence varicella-zoster virus (VZV) coinfections among confirmed mpox cases in Africa by country

**Study design**


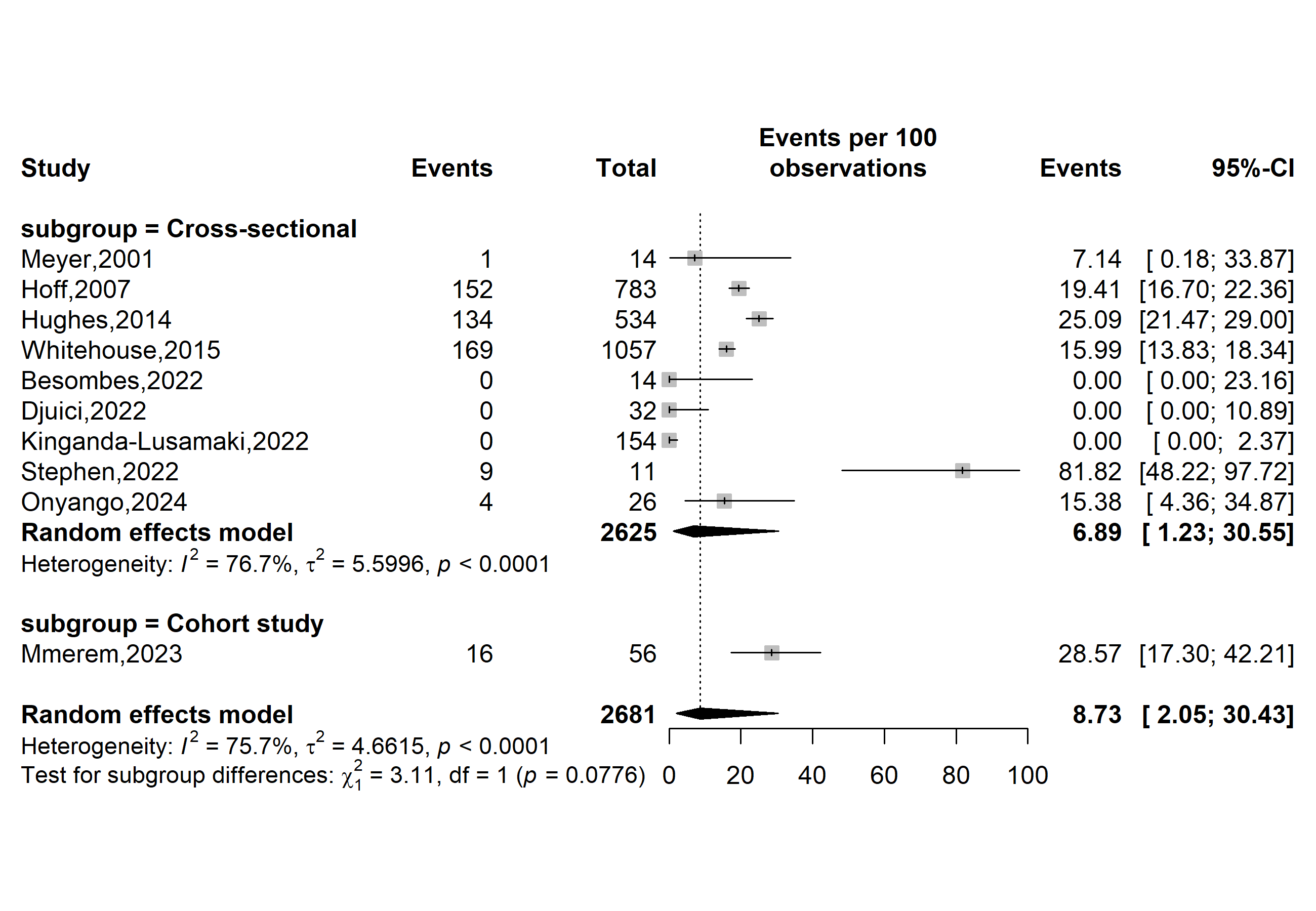


**Supplementary Fig. 3** Prevalence varicella-zoster virus (VZV) coinfections among confirmed mpox cases in Africa by design

**Study setting**


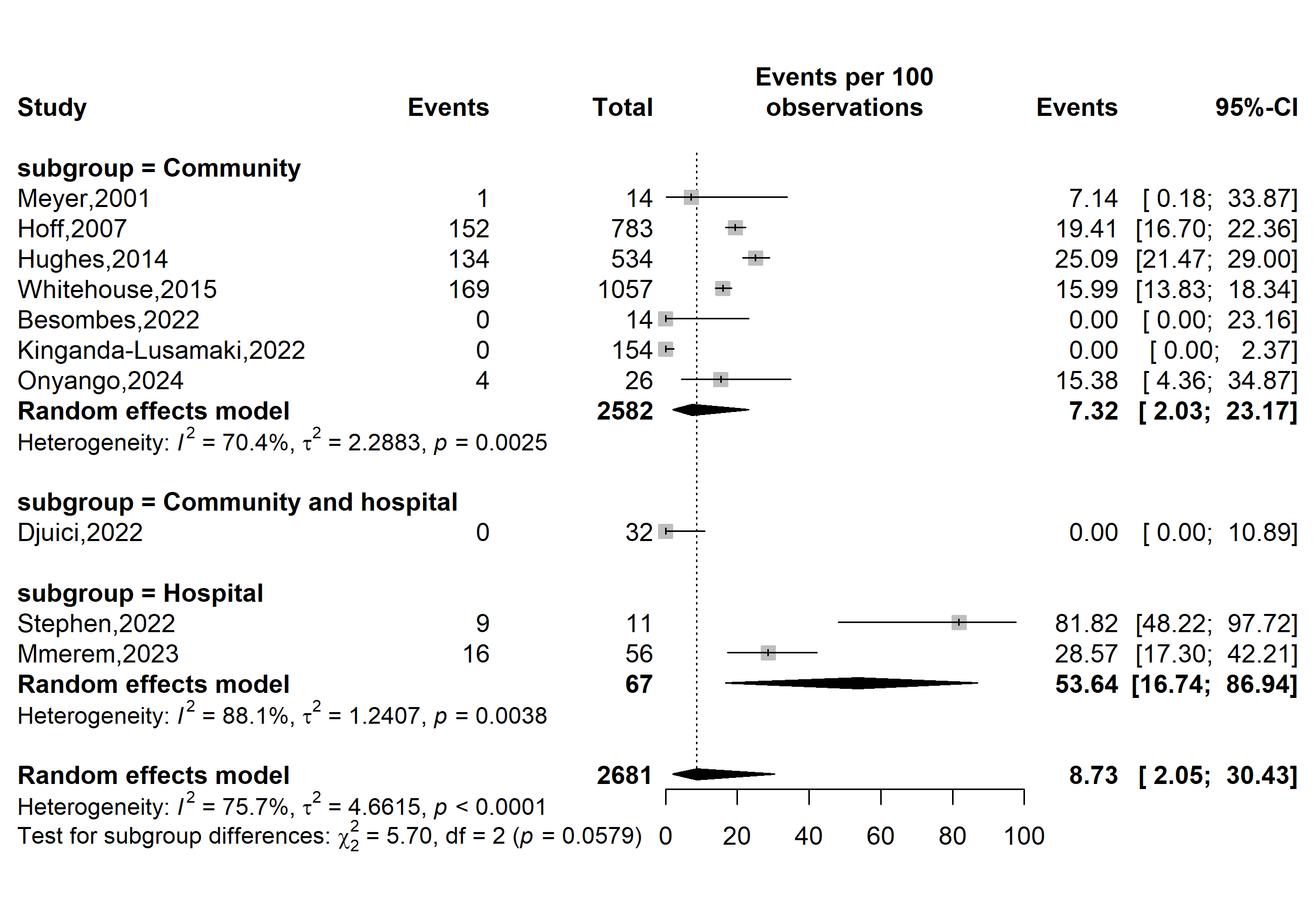


**Supplementary Fig. 4** Prevalence varicella-zoster virus (VZV) coinfections among confirmed mpox cases in Africa by study setting

**WHO Afro region**


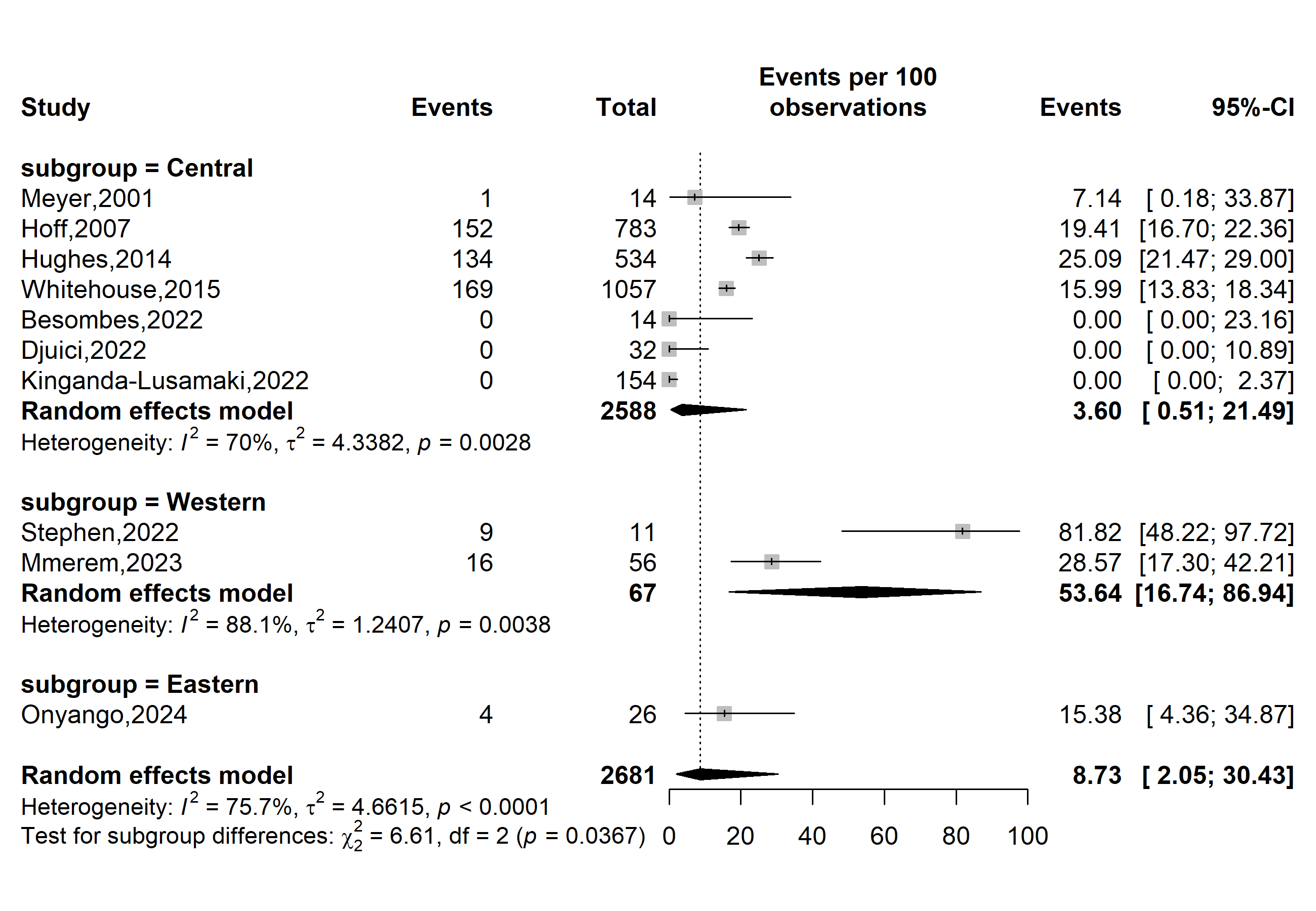


**Supplementary Fig. 5** Prevalence varicella-zoster virus (VZV) coinfections among confirmed mpox cases in Africa by WHO Afro region

**Publication bias**


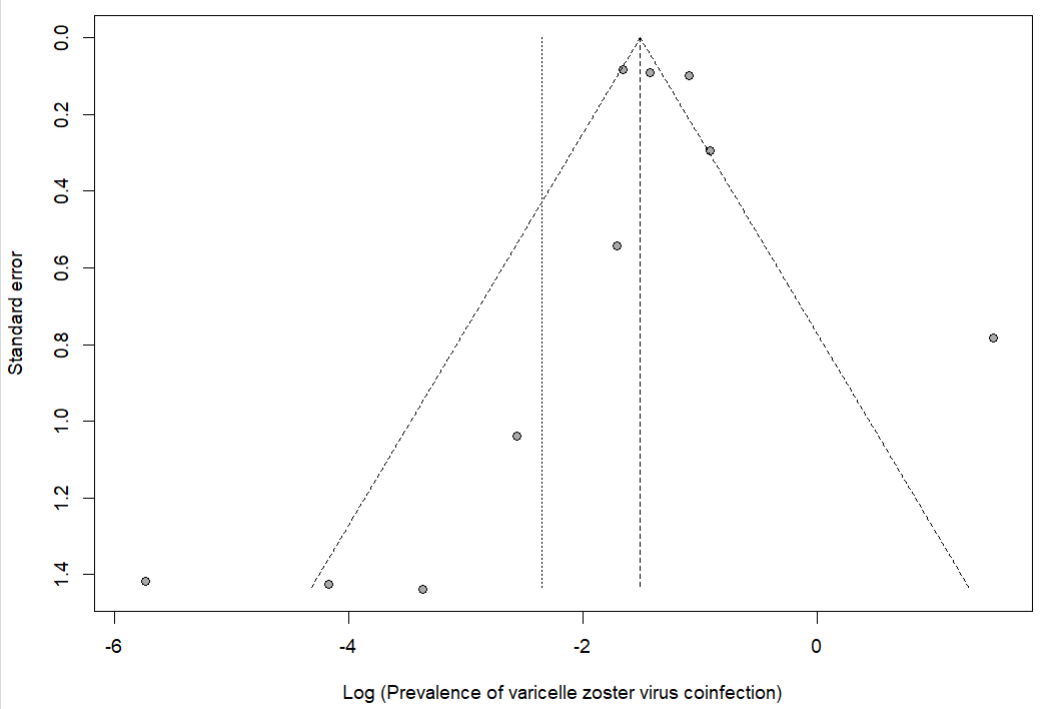


Egger’s test *p*-value = 0.689

Begg’s test *p*-value = 0.421

**Supplementary Fig. 6** Funnel plot displaying the pseudo 95% confidence limits and tests assessing the publication bias of studies included

**Sensitivity analysis**


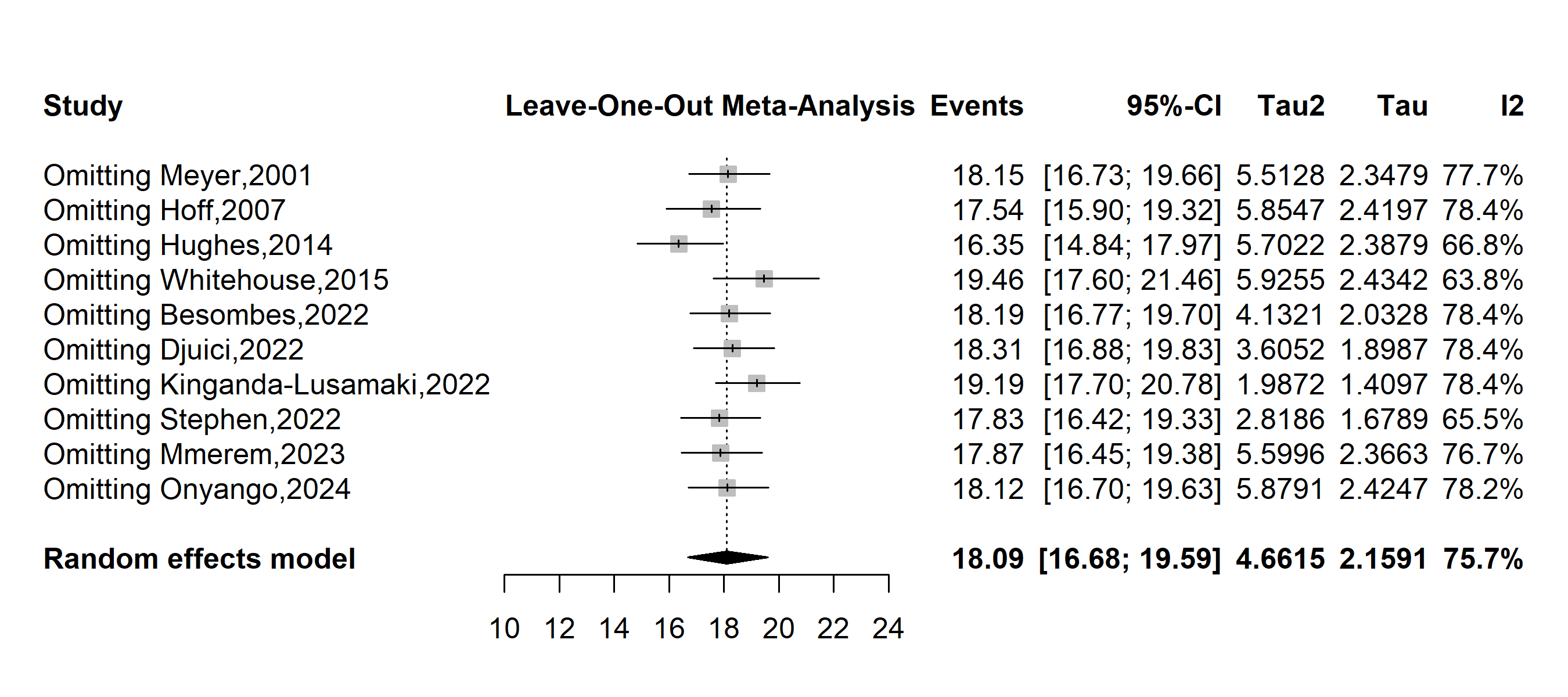


**Supplementary Fig. 7** Sensitivity analysis of the prevalence of varicella-zoster virus coinfections among confirmed mpox cases in Africa
