# Supplementary material for: Mpox coinfections and clinical manifestation in Africa: a systematic review and meta-analysis
Source: Front Syst Biol. 2026 May 7;6:1795422. doi: 10.3389/fsysb.2026.1795422 (PMC13189820; doi:10.3389/fsysb.2026.1795422)
Supplement: Supplementary file 2 [file Supplementaryfile3.docx]

**Study period**


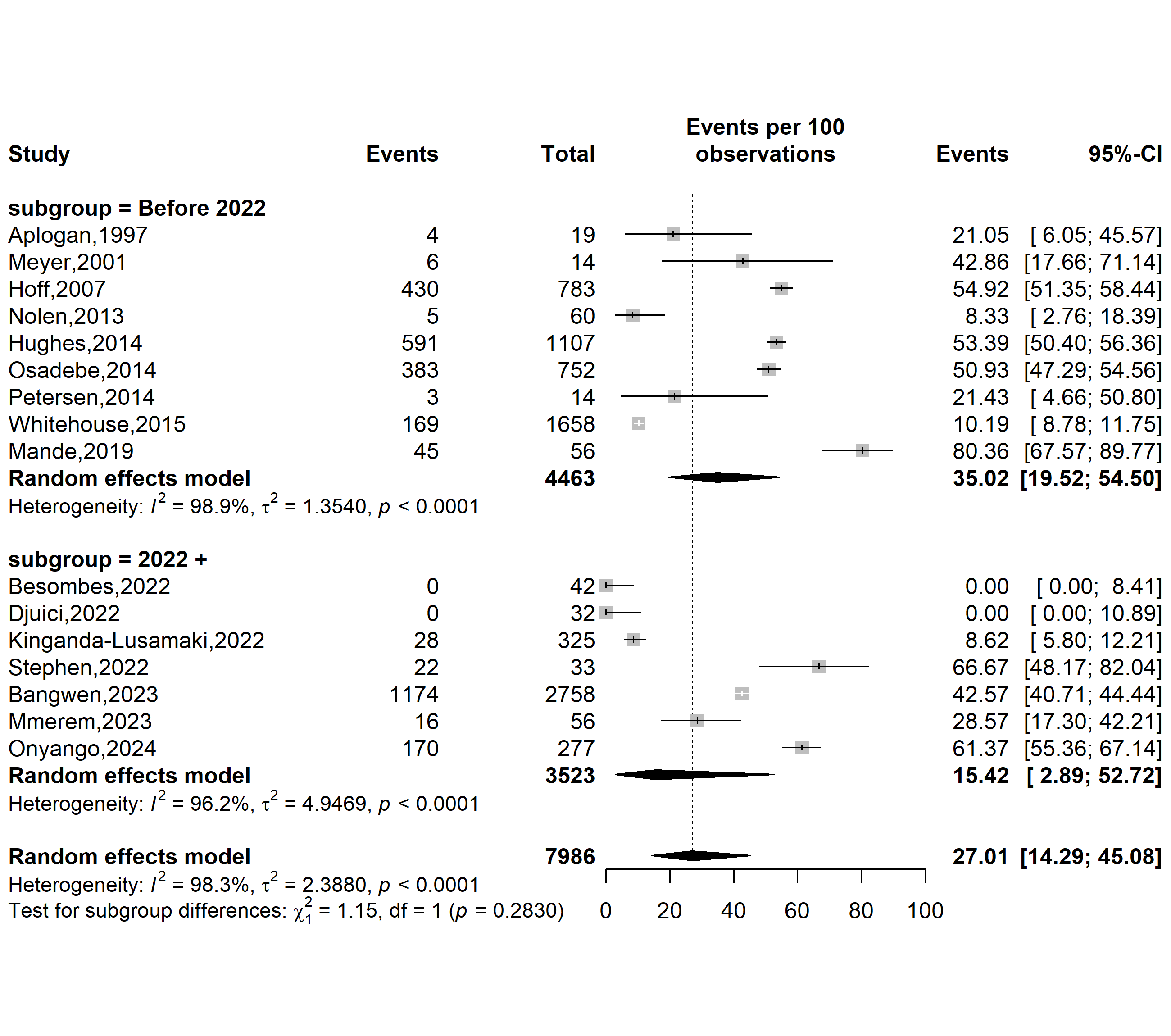


**Supplementary Fig. 1** Prevalence varicella-zoster virus infections in Africa by study period

**Country**


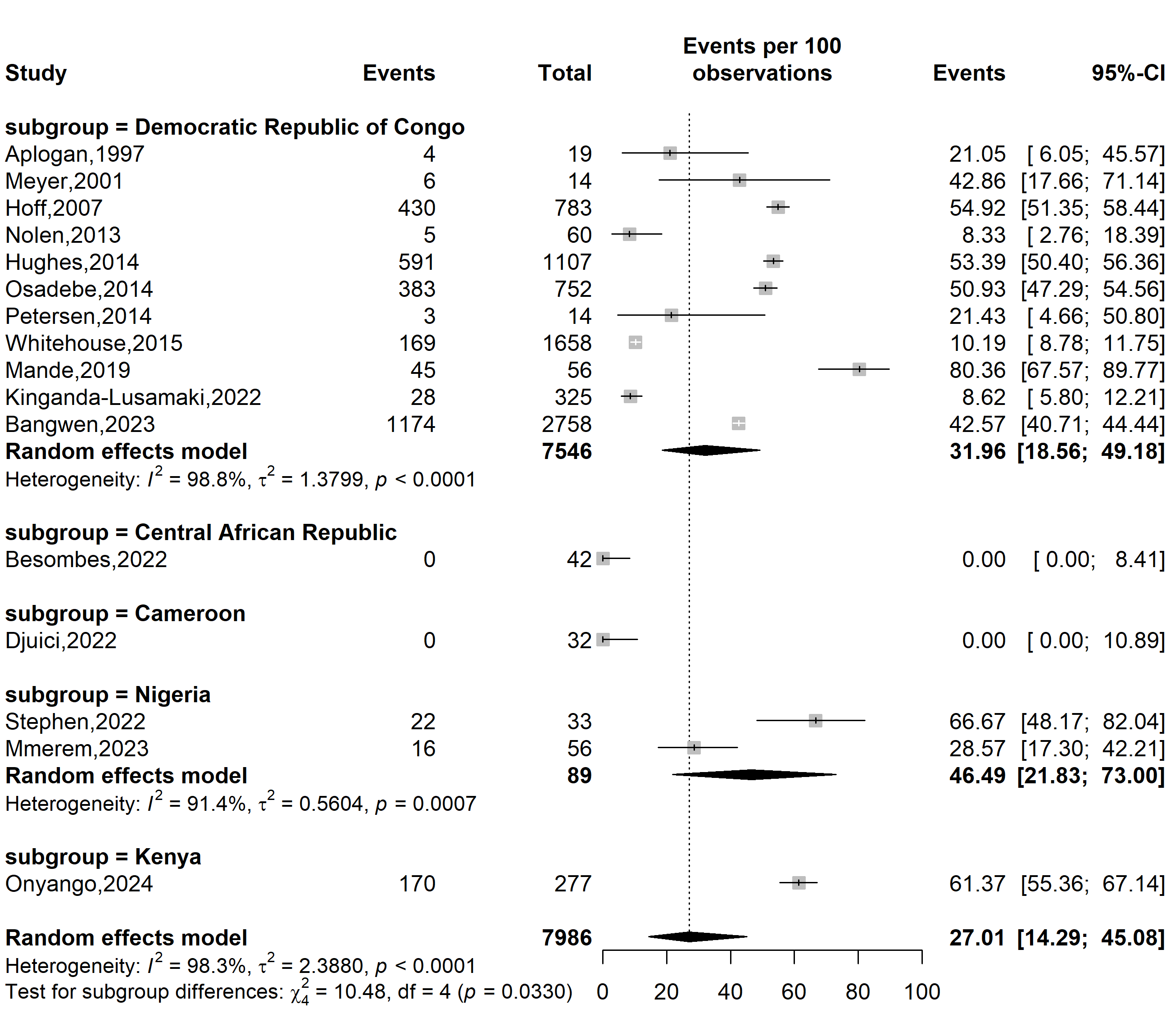


**Supplementary Fig. 2** Prevalence varicella-zoster virus infections in Africa by country

**Study design**


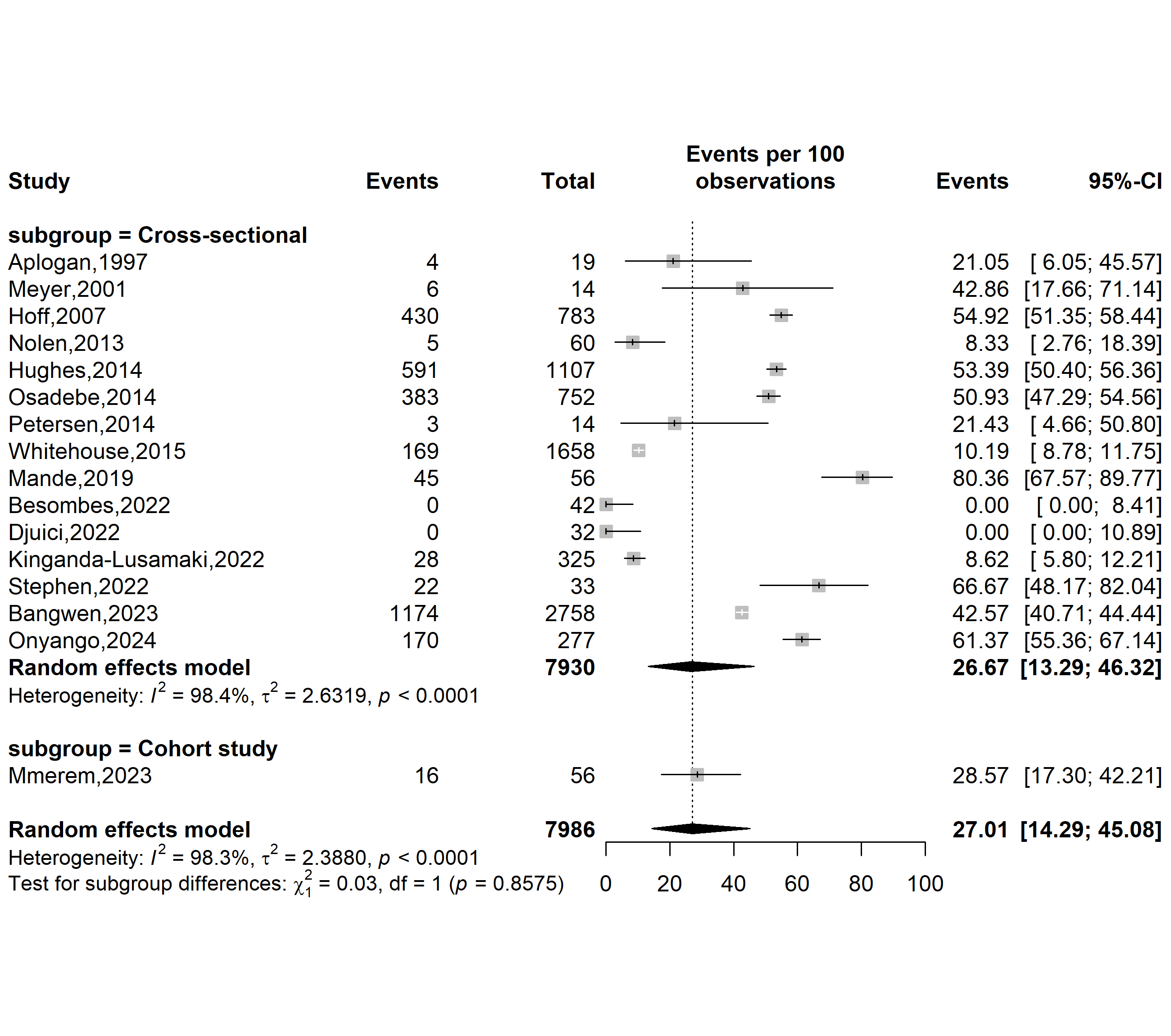


**Supplementary Fig. 3** Prevalence varicella-zoster virus infections in Africa by study design

**Study setting**


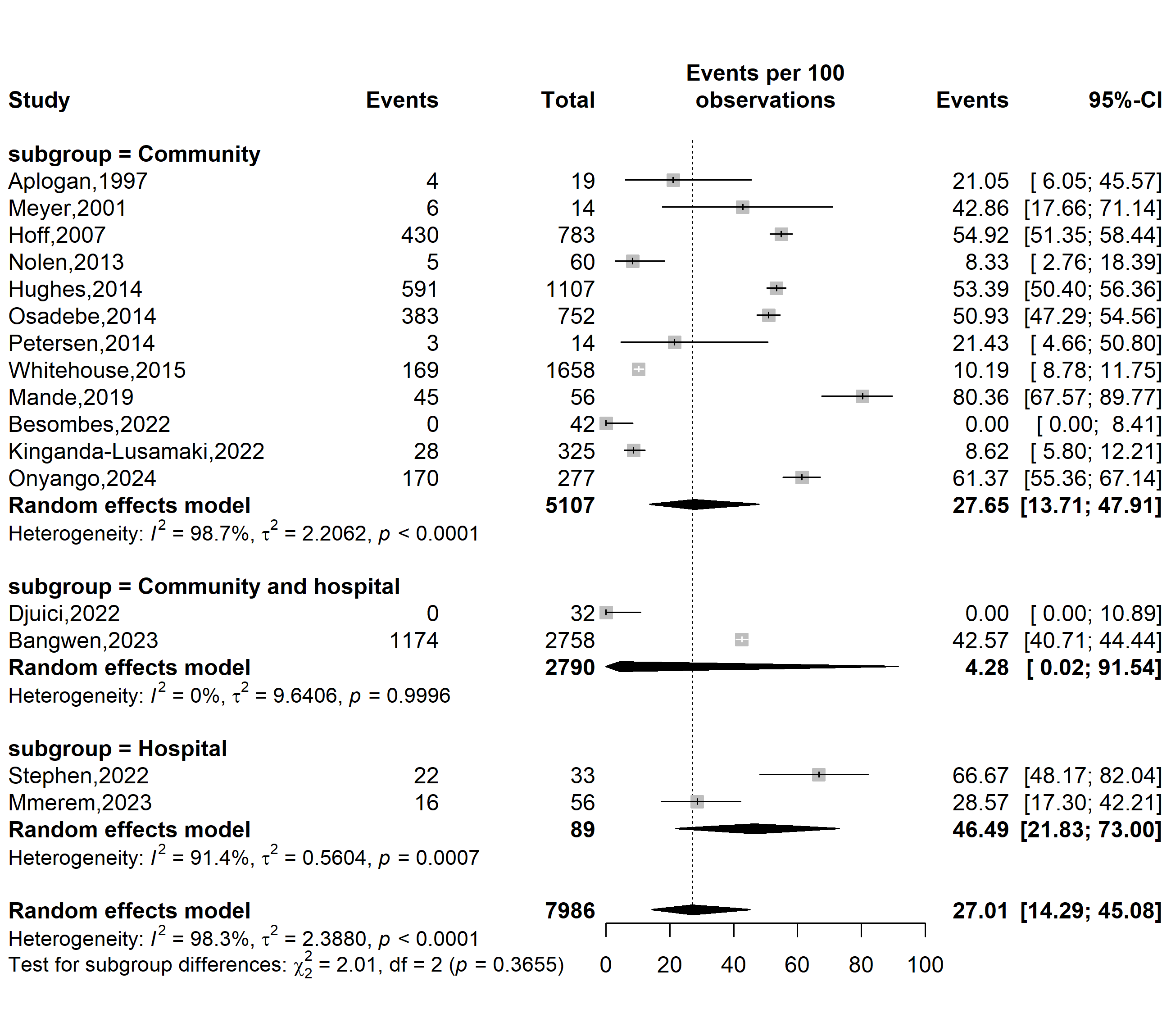


**Supplementary Fig. 4** Prevalence varicella-zoster virus infections in Africa by study setting

**Study participant**


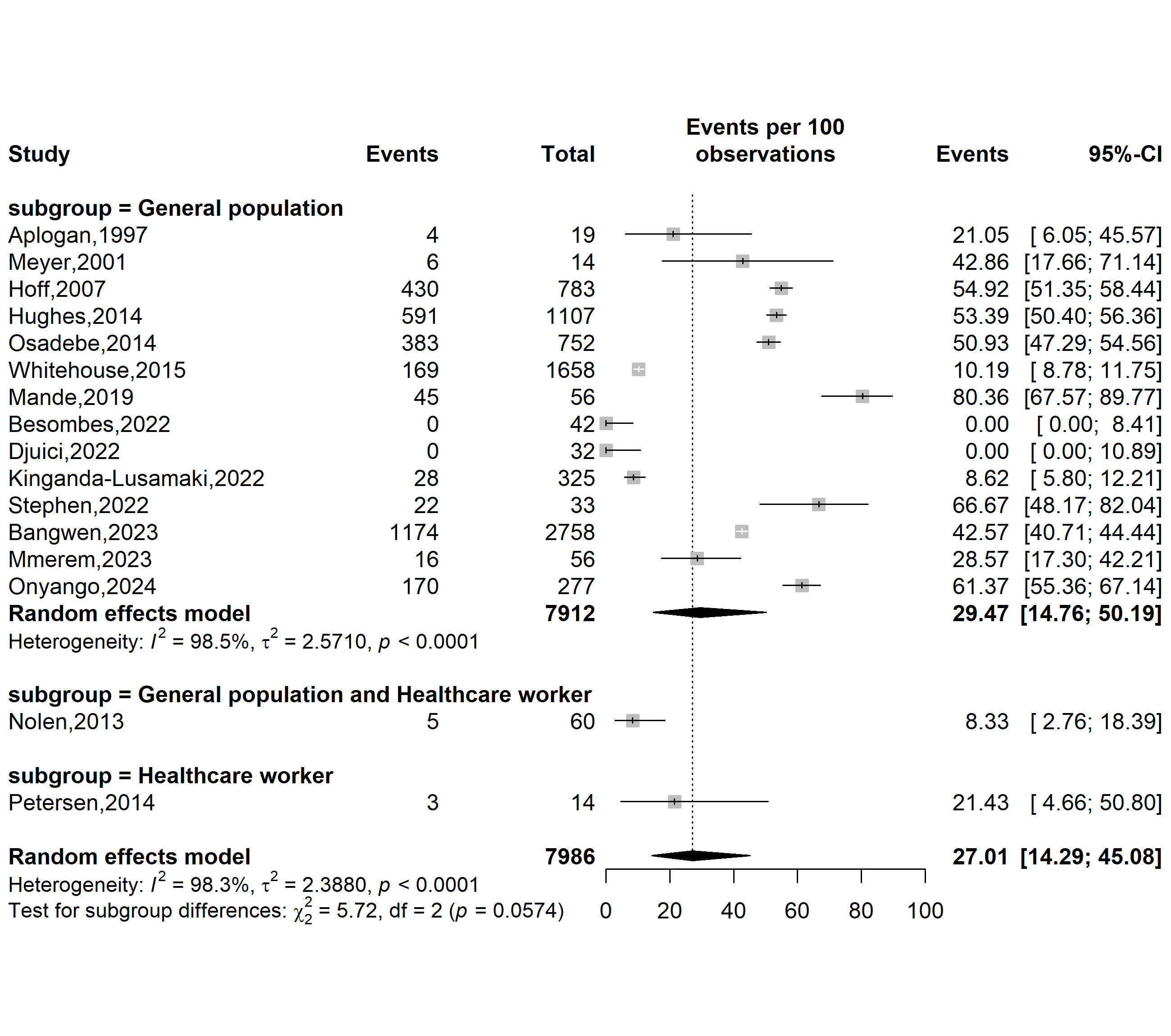


**Supplementary Fig. 5** Prevalence varicella-zoster virus in Africa by study participants

**Study WHO Afro region**


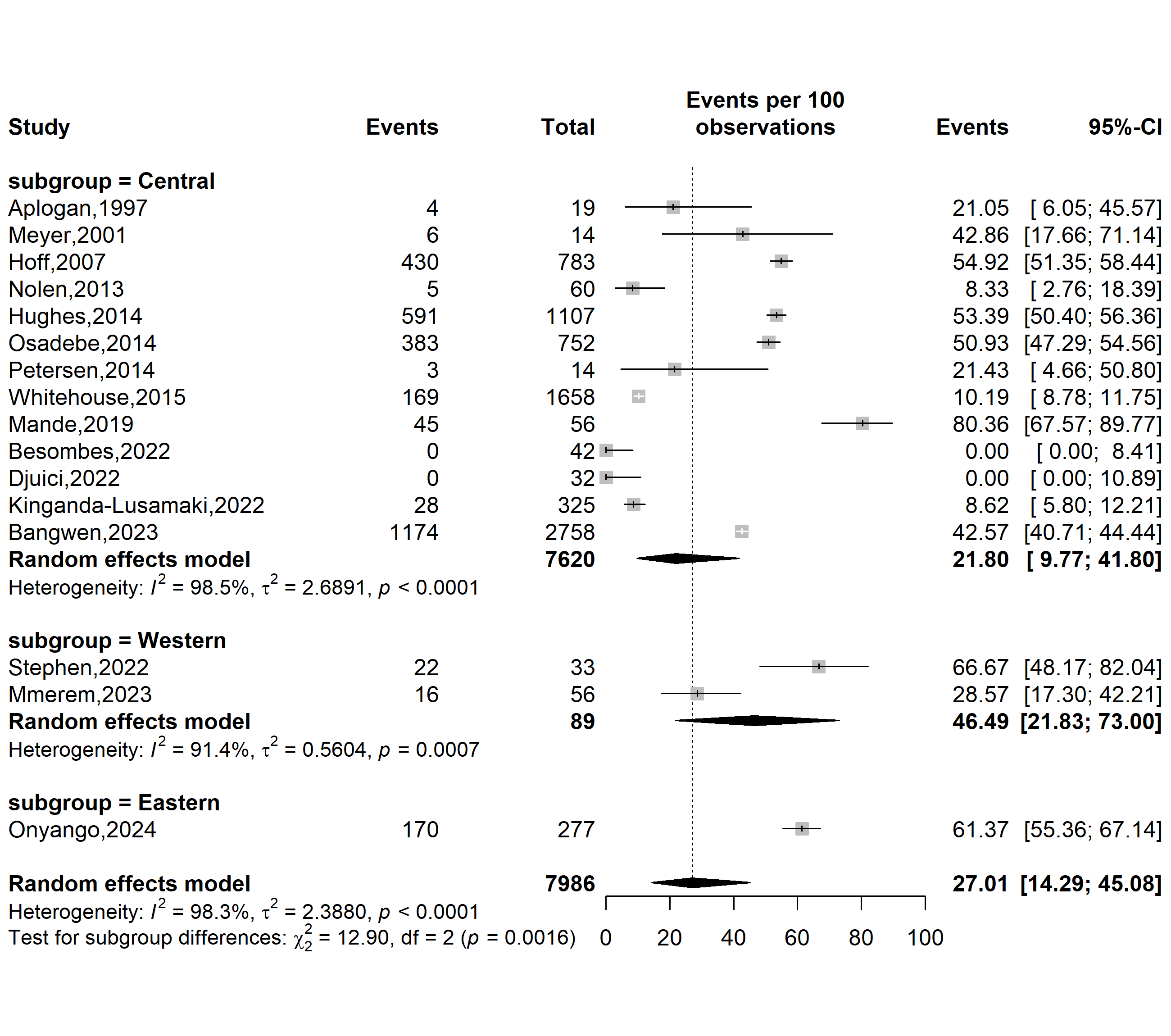


**Supplementary Fig. 6** Prevalence varicella-zoster virus in Africa by WHO Afro region

**Publication bias**

Egger’s test *p*-value = 0.493

Begg’s test *p*-value = 0.071


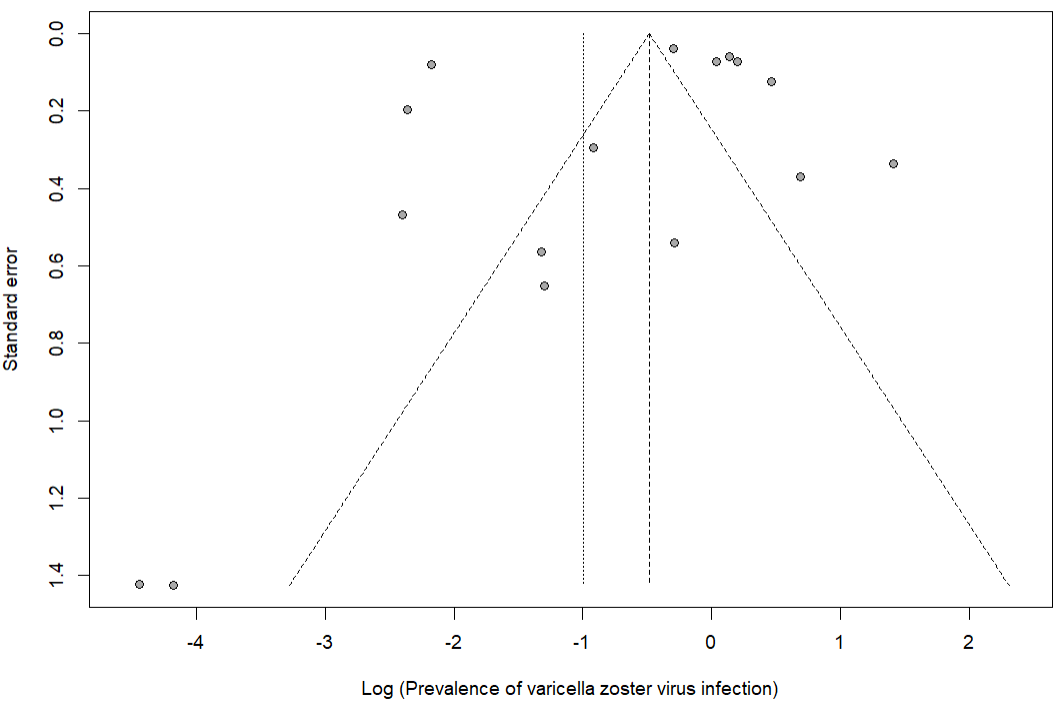


**Supplementary Fig. 7** Funnel plot displaying the pseudo 95% confidence limits and tests assessing the publication bias among studies included

**Sensitivity analysis**


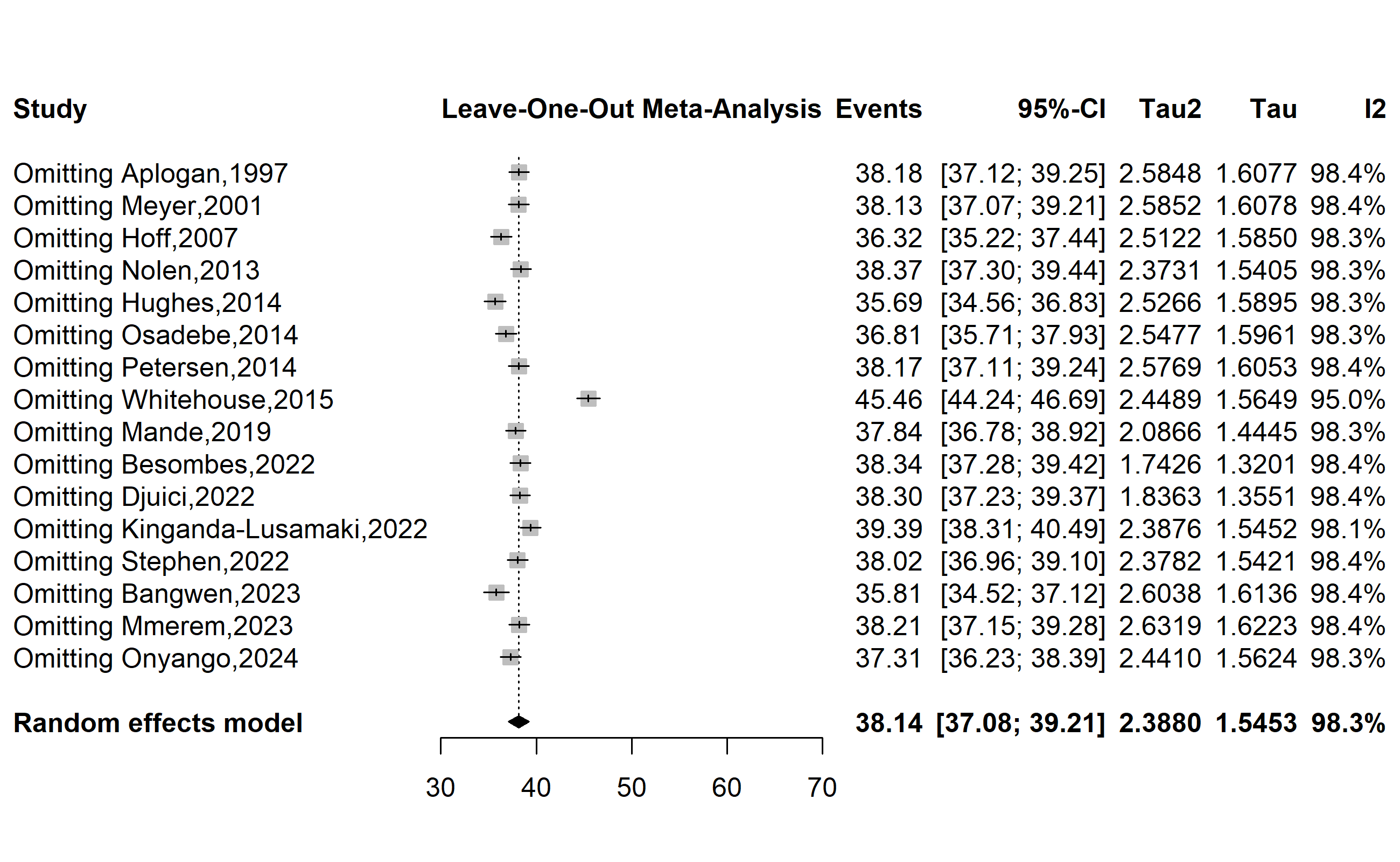


**Supplementary Fig. 8** Sensitivity analysis of the prevalence of varicella-zoster virus in Africa
