# Supplementary material for: Mpox coinfections and clinical manifestation in Africa: a systematic review and meta-analysis
Source: Front Syst Biol. 2026 May 7;6:1795422. doi: 10.3389/fsysb.2026.1795422 (PMC13189820; doi:10.3389/fsysb.2026.1795422)
Supplement: Supplementary file 3 [file Supplementaryfile2.docx]

**Study period**


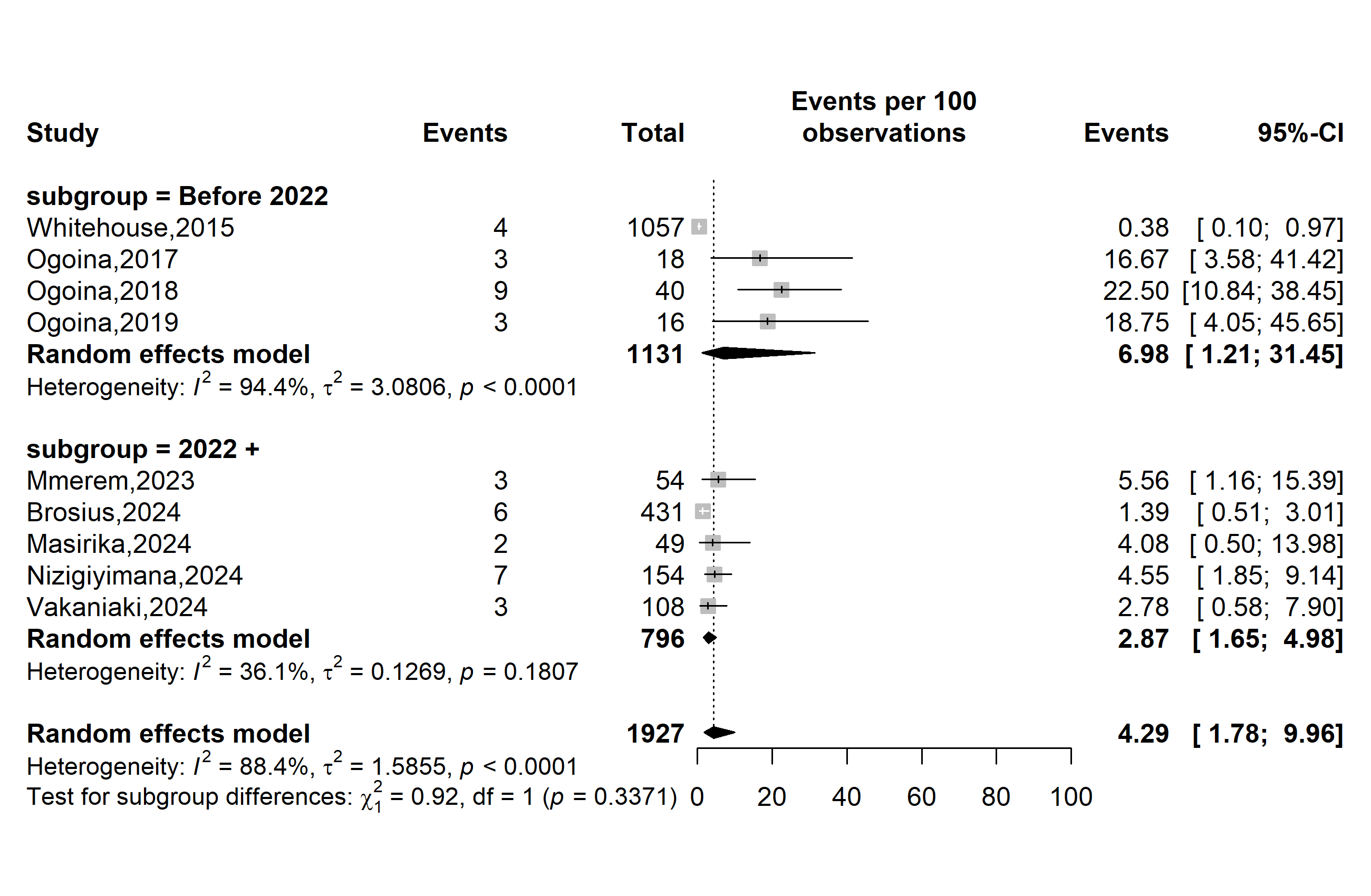


**Supplementary Fig. 1** Prevalence of human immunodeficiency virus (HIV) coinfections among confirmed mpox cases in Africa by study period

**Country**


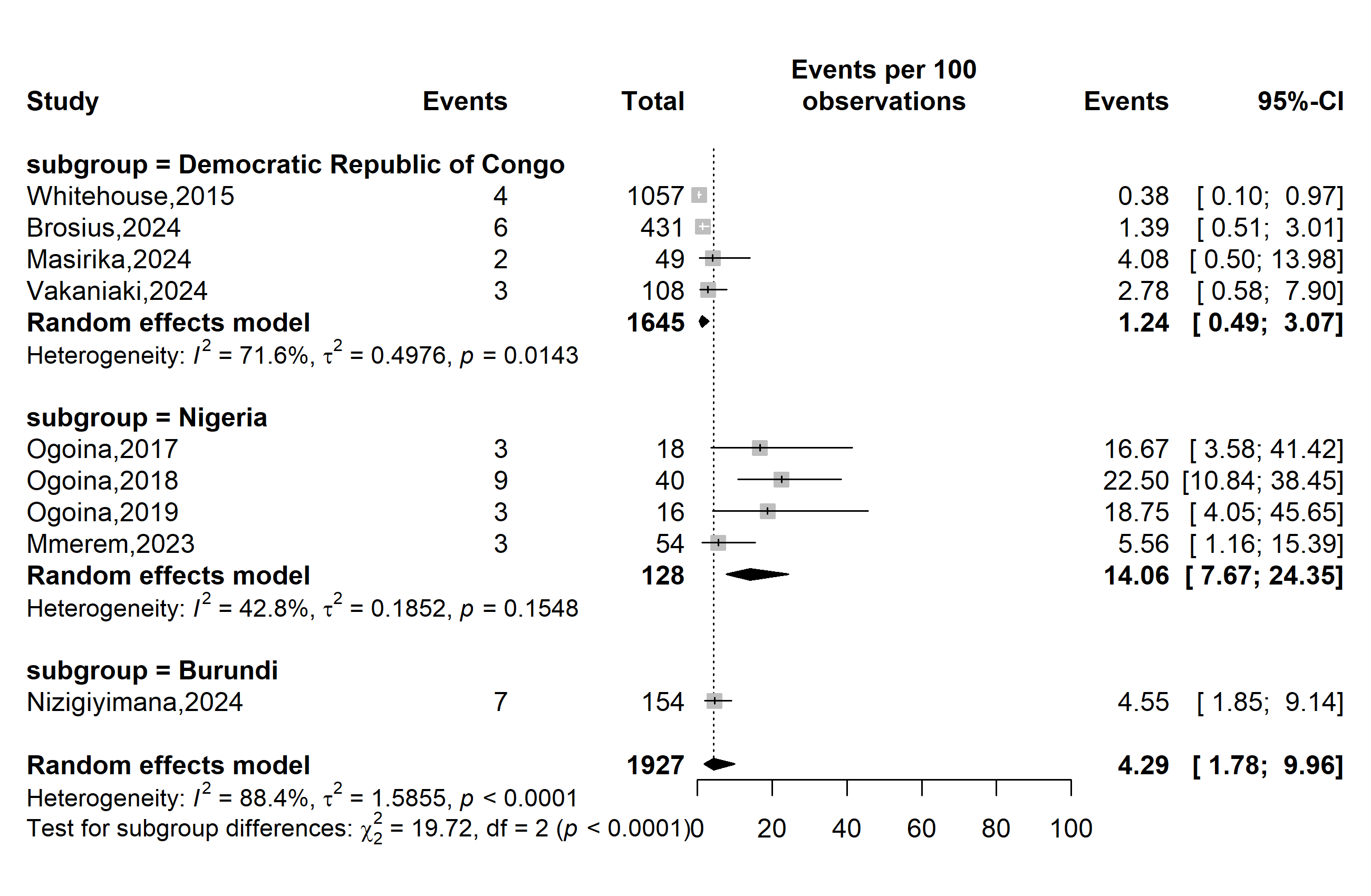


**Supplementary Fig. 2** Prevalence of human immunodeficiency virus (HIV) coinfections among confirmed mpox cases in Africa by country

**Study design**


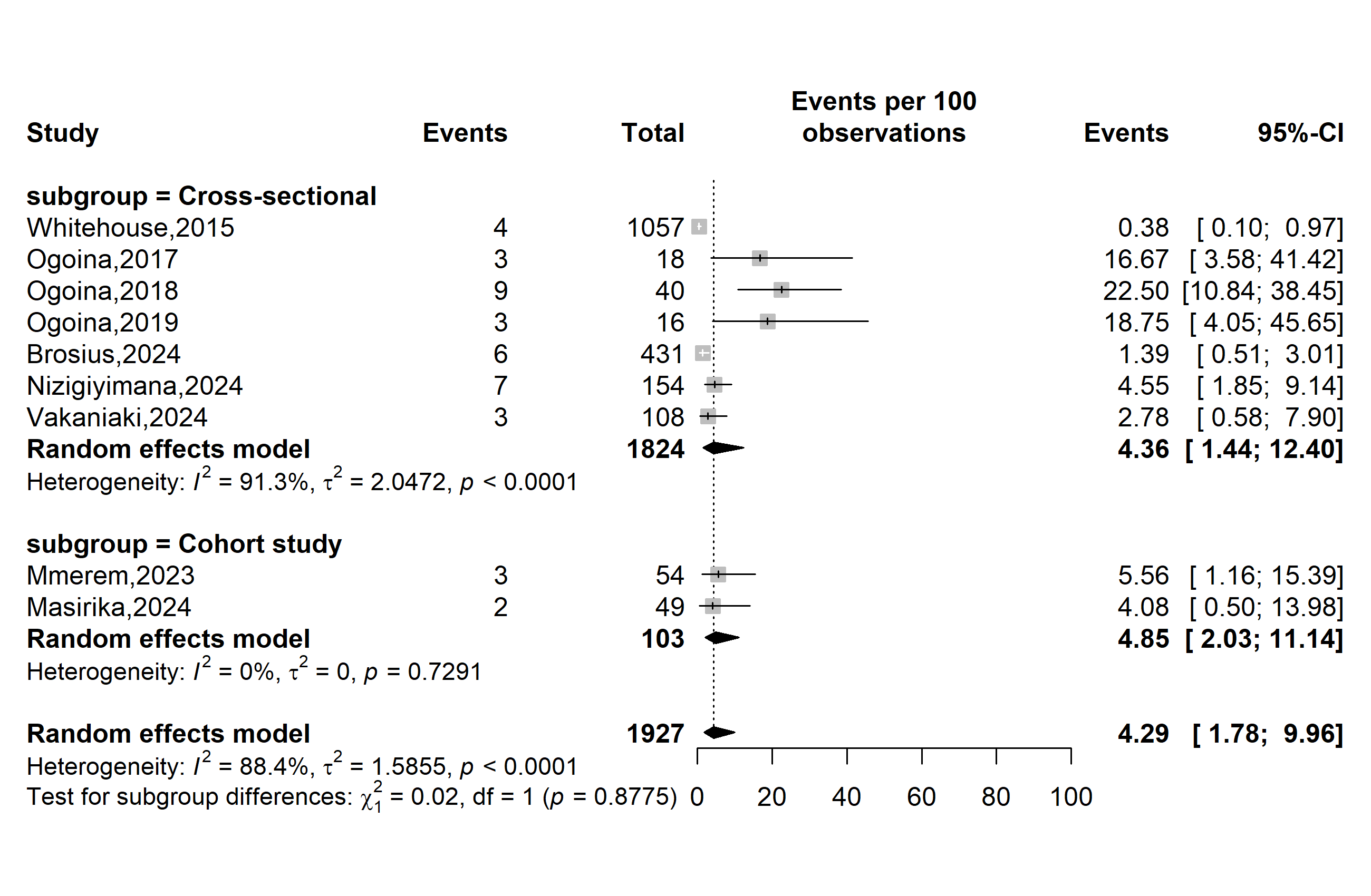


**Supplementary Fig. 3** Prevalence of human immunodeficiency virus (HIV) coinfections among confirmed mpox cases in Africa by study design

**Study setting**


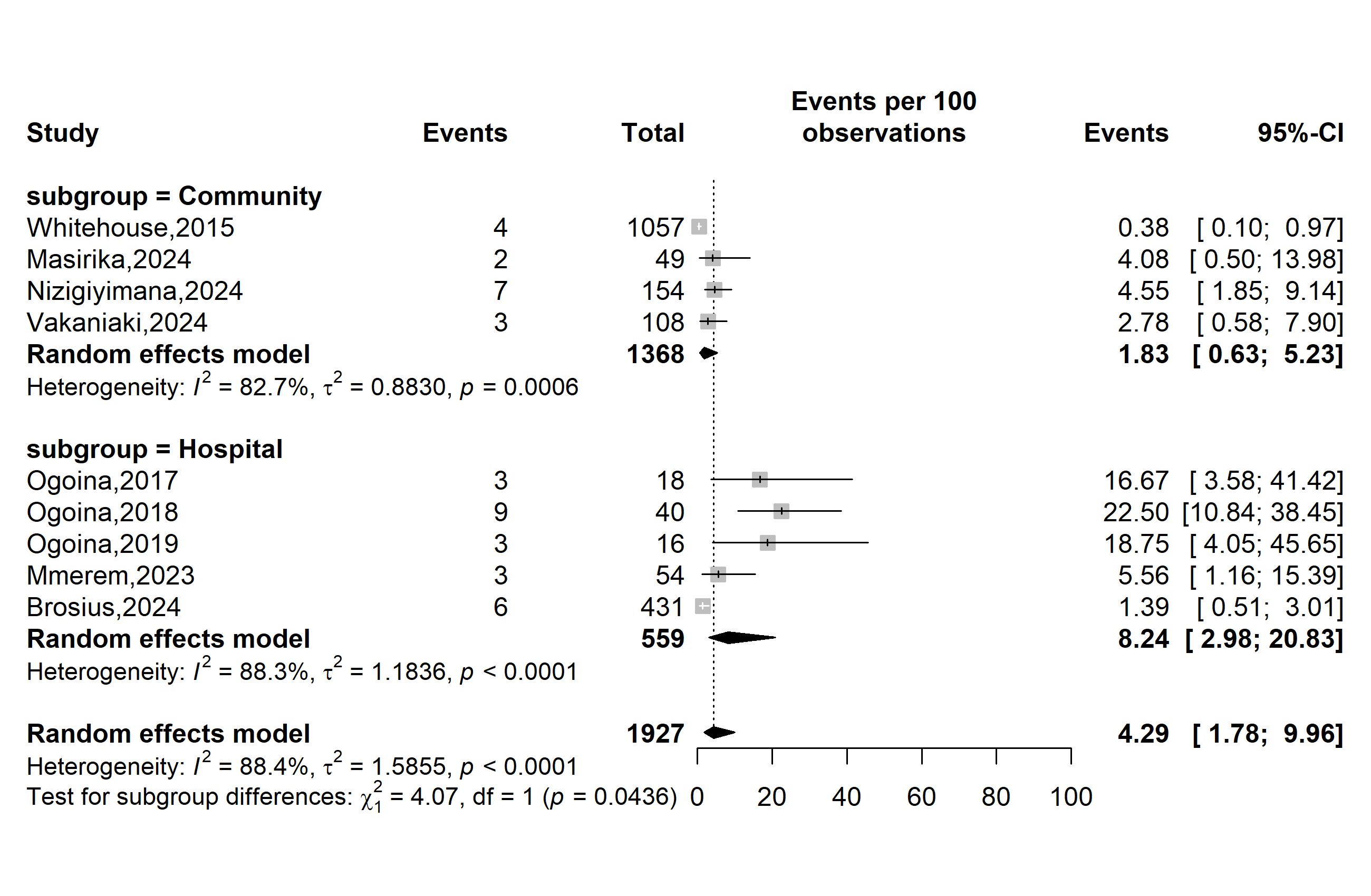


**Supplementary Fig. 4** Prevalence of human immunodeficiency virus (HIV) coinfections among confirmed mpox cases in Africa by study setting

**WHO Afro region**


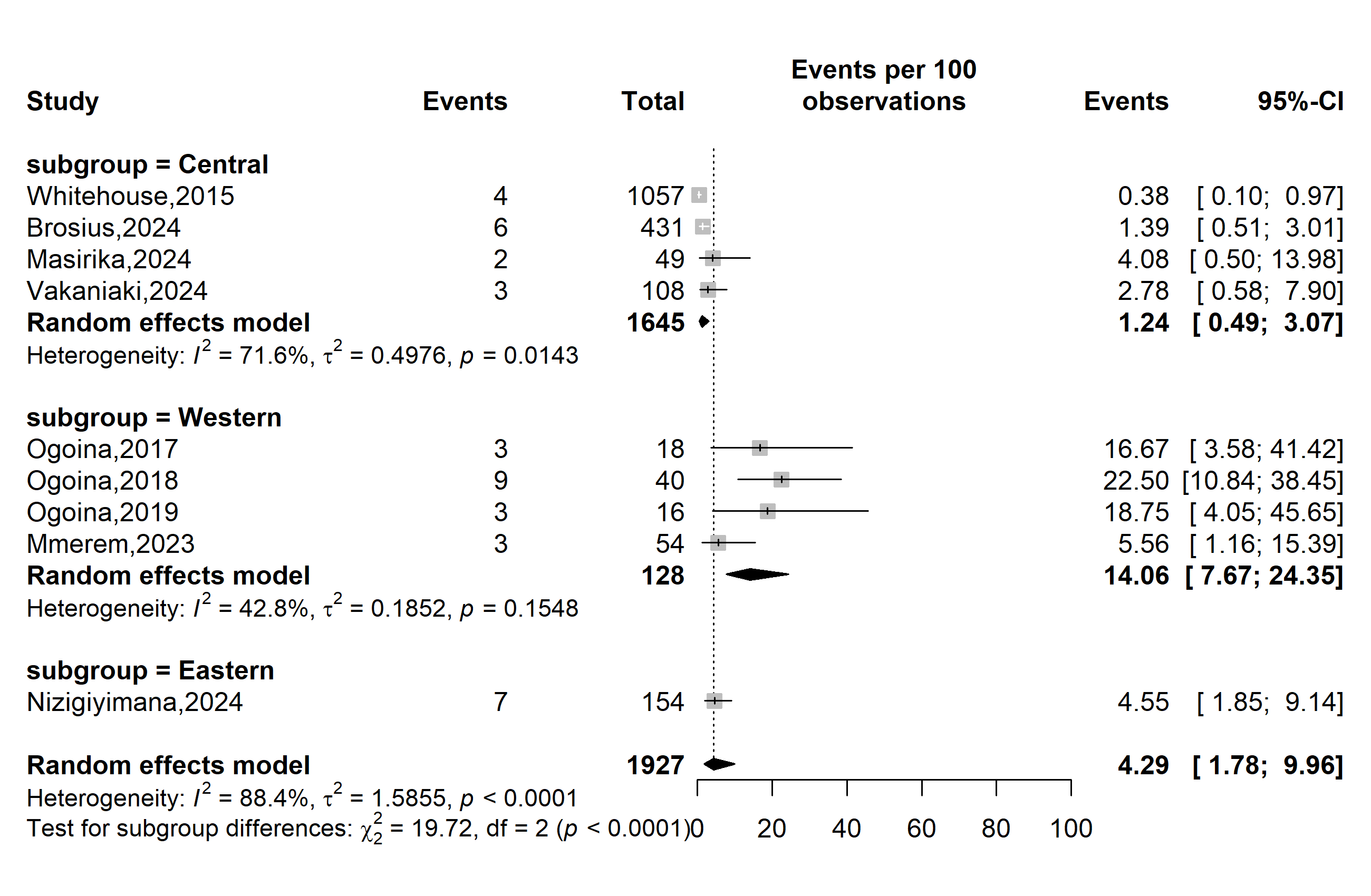


**Supplementary Fig. 5** Prevalence of human immunodeficiency virus (HIV) coinfections among confirmed mpox cases in Africa by WHO Afro region

**Publication bias**


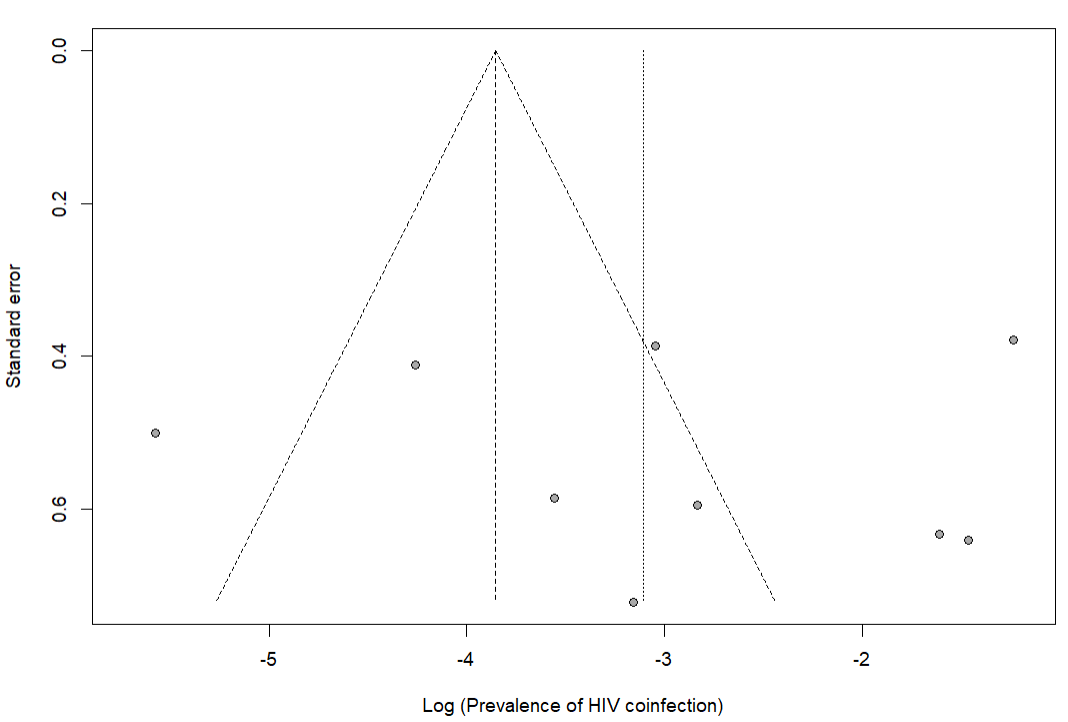


**Supplementary Fig. 6** Funnel plot assessing the risk of publication bias among studies on the prevalence of human immunodeficiency virus (HIV) infections among confirmed mpox cases in Africa


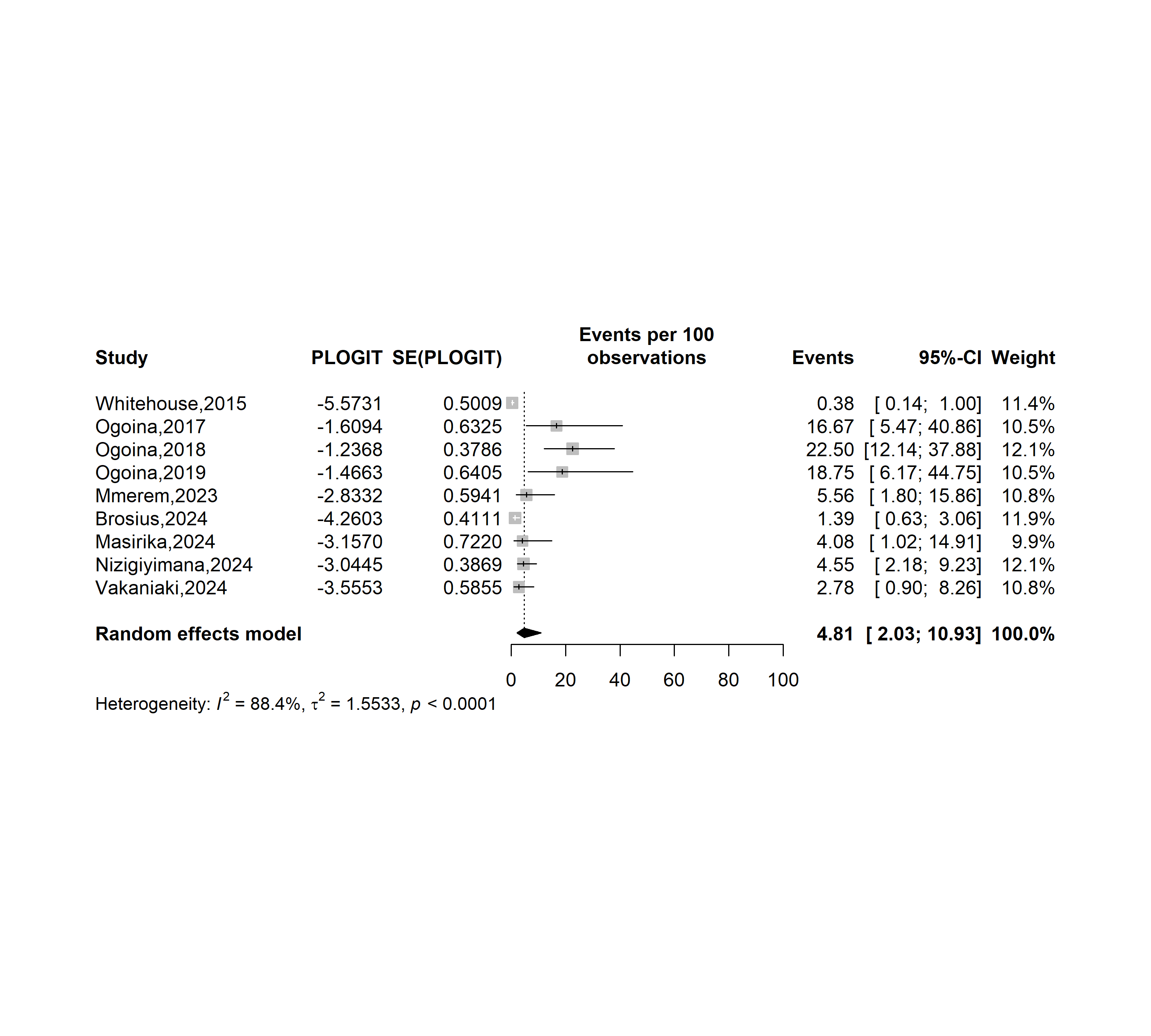


**Supplementary Fig. 7** Trim-and-fill analysis adjusting the prevalence of human immunodeficiency virus (HIV) infections among confirmed mpox cases in Africa

**Sensitivity analysis**


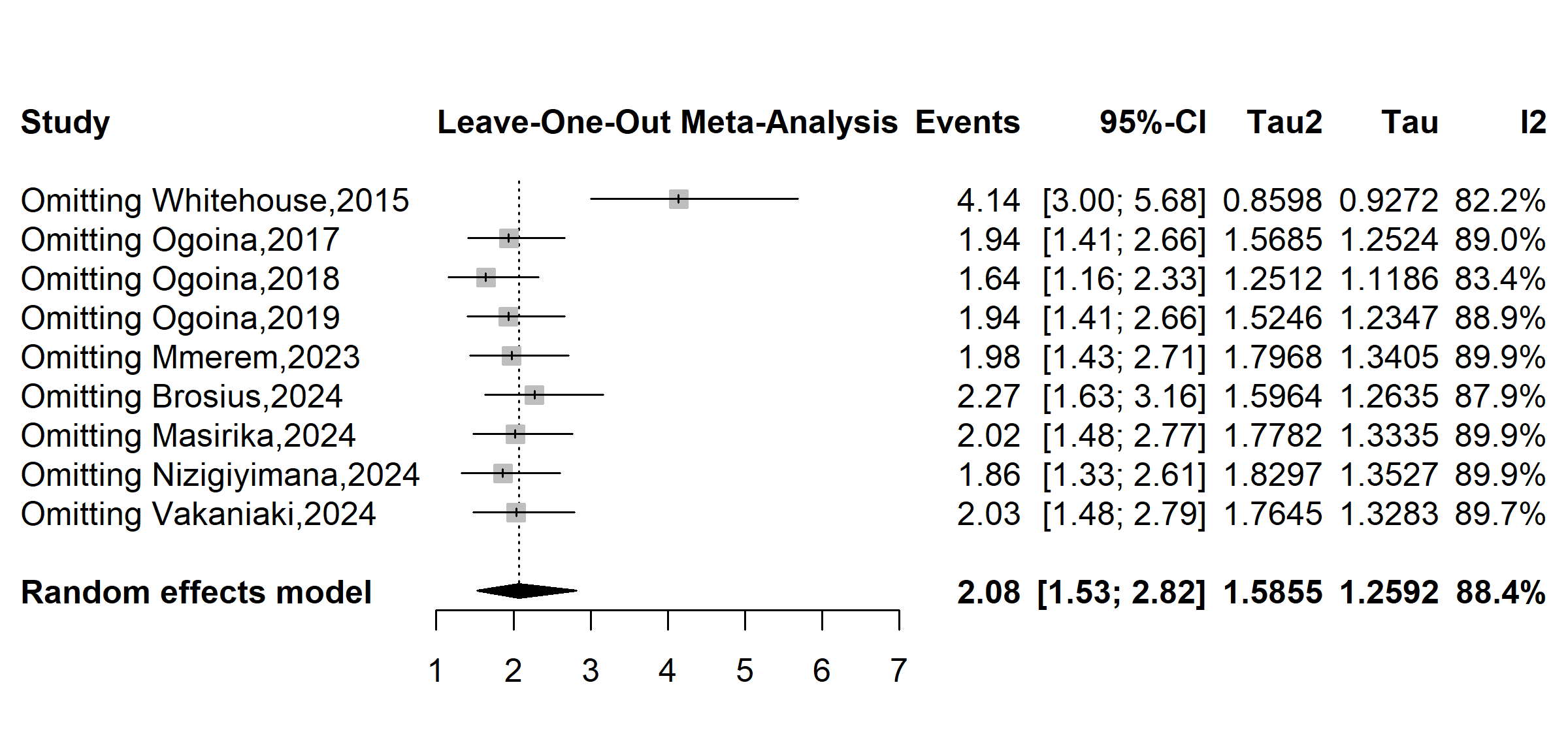


**Supplementary Fig. 8** Sensitivity analysis of the Prevalence of human immunodeficiency virus (HIV) coinfections among confirmed mpox cases in Africa
