# Supplementary material for: Mpox coinfections and clinical manifestation in Africa: a systematic review and meta-analysis
Source: Front Syst Biol. 2026 May 7;6:1795422. doi: 10.3389/fsysb.2026.1795422 (PMC13189820; doi:10.3389/fsysb.2026.1795422)
Supplement: Supplementary file 4 [file Supplementaryfile4.docx]

**Clade I**

**Supplementary Fig. 1** Descriptive narrative synthesis of clinical manifestations of Mpox disease by viral strains in Africa

**Supplementary Table 1** Clinical pattern of confirmed mpox cases by genotypic category in Africa

| **Clinical manifestation** | **Genotypic classification of circulating mpox virus (Clade)**  ***n* (%)** | | | | | | | | | | ***p*-value^1^** |
| --- | --- | --- | --- | --- | --- | --- | --- | --- | --- | --- | --- |
|  | **I** | | **Ia** | | **Ib** | | **II** | | **I and II** | |  |
|  | Total | *n* (%) | Total | *n* (%) | Total | *n* (%) | Total | *n* (%) | Total | *n* (%) |  |
| Rash | 10 | 10 (100.0) | 14 | 14 (100.0) | 547 | 534 (97.6) | 139 | 139 (100.0) | 30 | 29 (96.7) | 0.118 |
| Fever | 10 | 10 (100.0) | 15 | 13 (86.7) | 541 | 91 (16.8) | 113 | 100 (88.5) | 26 | 22 (84.6) | < 0.001*** |
| Lymphadenopathy | 10 | 10 (100.0) | 10 | 9 (90.0) | 531 | 347 (65.3) | 86 | 58 (67.4) | 26 | 12 (46.2) | < 0.001*** |
| Sore throat or dysphagia | 10 | 10 (100.0) | 9 | 6 (66.7) | 534 | 262 (49.1) | 77 | 45 (58.4) | 26 | 16 (61.5) | < 0.001*** |
| Myalgia | 10 | 8 (80.0) | 9 | 9 (100.0) | 543 | 329 (60.6) | 88 | 47 (53.4) | 26 | 9 (34.6) | < 0.001*** |
| Oral lesions | 10 | 7 (70.0) | 15 | 10 (66.7) | 528 | 121 (22.9) | 139 | 45 (32.4) | 26 | 11 (42.3) | < 0.001*** |
| Fatigue | 10 | 6 (60.0) | 10 | 10 (100.0) | 537 | 403 (75.0) | 118 | 61 (51.7) | 27 | 20 (74.1) | < 0.001*** |
| Headache | 10 | 5 (50.0) | 9 | 8 (88.9) | 539 | 275 (51.0) | 98 | 73 (74.5) | 25 | 15 (60.0) | 0.002** |
| Cough | 10 | 5 (50.0) | 10 | 7 (70.0) | 541 | 171 (31.6) | 139 | 37 (26.6) | 27 | 13 (48.1) | < 0.001*** |
| Conjunctivitis | 10 | 5 (50.0) | 10 | 6 (60.0) | 423 | 16 (3.8) | 139 | 31 (22.3) | 26 | 2 (7.7) | < 0.001*** |
| Palm lesions | 10 | 5 (50.0) | 10 | 9 (90.0) | 431 | 167 (38.7) | 70 | 48 (68.6) | - | - | < 0.001*** |
| Anorexia | 10 | 5 (50.0) | - | - | 429 | 244 (56.9) | 21 | 2 (9.5) | - | - | < 0.001*** |
| Sole lesions | 10 | 4 (40.0) | 9 | 8 (88.9) | 431 | 100 (23.2) | 66 | 42 (63.6) | - | - | < 0.001*** |
| Chills or sweat | - | - | 10 | 9 (90.0) | 9 | 1 (11.1) | 139 | 87 (62.6) | 27 | 18 (66.7) | < 0.001*** |
| Pruritus or itchy lesion | - | - | 15 | 14 (93.3) | 426 | 390 (91.5) | 99 | 71 (71.7) | 27 | 19 (70.4) | < 0.001*** |
| Vomiting or nausea | - | - | 10 | 2 (20.0) | 428 | 97 (22.7) | 139 | 29 (20.9) | 26 | 4 (15.4) | 0.925 |
| Genital lesions | - | - | 14 | 13 (92.9) | 428 | 340 (79.4) | 86 | 57 (66.3) | - | - | 0.001** |
| Dyspnea | 10 | 6 (60.0) | - | - | 428 | 59 (13.8) | - | - | - | - | < 0.001 |
| Abdominal pain | 10 | 4 (40.0) | - | - | 424 | 128 (30.2) | - | - | - | - | 0.584 |
| Light sensitivity | - | - | 9 | 8 (88.9) | - | - | 139 | 29 (20.9) | - | - | < 0.001*** |
| Diarrhea | - | - | 9 | 1 (11.1) | 437 | 55 (12.6) | 21 | 1 (4.8) | - | - | 0.723 |
| Malaise | - | - |  |  | 428 | 320 (74.8) | 21 | 13 (61.9) | - | - | 0.177 |
| Bedridden status | - | - | 10 | 2 (20.0) | 108 | 10 (9.3) | - | - | - | - | 0.307 |
| Hemorrhagic skin lesions | - | - | 9 | 2 (22.2) | - | - | - | - | - | - | - |
| Painful lesion | - | - | - | - | 426 | 333 (78.2) | - | - | - | - | - |
| Dysuria | - | - | - | - | 425 | 168 (39.5) | - | - | - | - | - |
| Anal lesions | - | - | - | - | 431 | 136 (31.6) | - | - | - | - | - |
| Painful eye | - | - | - | - | 426 | 85 (20.0) | - | - | - | - | - |
| Rectal pain | - | - | - | - | 424 | 46 (10.8) | - | - | - | - | - |
| Visual problem | - | - | - | - | 426 | 42 (9.9) | - | - | - | - | - |
| Hematuria | - | - | - | - | 428 | 22 (5.1) | - | - | - | - | - |
| Confusion | - | - | - | - | 429 | 12 (2.8) | - | - | - | - | - |
| Convulsions | - | - | - | - | 429 | 1 (0.2) | - | - | - | - | - |
| Joint pain | - | - | - | - | - | - | 21 | 5 (23.8) | - | - | - |
| Dehydration | - | - | - | - | - | - | 21 | 2 (9.5) | - | - | - |
| Hepatomegaly | - | - | - | - | - | - | 21 | 2 (9.5) | - | - | - |
| Scrotal edema | - | - | - | - | - | - | 21 | 1 (4.8) | - | - | - |
| Number of studies | 1 | | 2 | | 3 | | 2 | | 1 | |  |
| Country [Reference] | Sudan (Formenty et al., 2010) | | CAR and DRC (Besombes et al., 2023; Kibungu et al., 2024) | | DRC (Mukadi-Bamuleka et al., 2024; Vakaniaki et al., 2024; Brosius et al., 2025) | | Nigeria (Ogoina et al., 2019; Yinka-Ogunleye et al., 2019a) | | Cameroon (Djuicy et al., 2024) | |  |
| ^1^ *Chi square or Fisher exact test; CAR: Central African Republic; DRC: Democratic Republic of Congo*; ****p < 0.001; **p < 0.01; *p < 0.05* | | | | | | | | | | | |

**Supplementary Table 2** Grading of Recommendations Assessment, Development and Evaluation for our study findings

| **Outcome** | **Risk of Bias** | **Inconsistency** | **Indirectness** | **Imprecision** | **Publication Bias** | **Overall Certainty** |
| --- | --- | --- | --- | --- | --- | --- |
| VZV–Mpox coinfection prevalence | Not serious | Serious (−1) | Not serious | Serious (−1) | Not detected | **Low** |
| HIV–Mpox coinfection prevalence | Not serious | Serious (−1) | Not serious | Serious (−1) | Not detected | **Low** |
| VZV infection prevalence | Not serious | Serious (−1) | Not serious | Serious (−1) | Not detected | **Low** |
| *VZV: Varicella-zoster virus; HIV: Human immunodeficiency virus* | | | | | | |
